# Comparative analysis of disease modelling for health economic evaluations of systemic therapies in advanced hepatocellular carcinoma

**DOI:** 10.1371/journal.pone.0292239

**Published:** 2023-10-05

**Authors:** Huimin Zou, Yan Xue, Xianwen Chen, Yunfeng Lai, Dongning Yao, Carolina Oi Lam Ung, Hao Hu

**Affiliations:** 1 State Key Laboratory of Quality Research in Chinese Medicine, Institute of Chinese Medical Sciences, University of Macau, Macao SAR, China; 2 School of Public Health and Management, Guangzhou University of Chinese Medicine, Guangzhou, China; 3 Department of Drug Regulatory Science and Pharmacoeconomics, School of Pharmacy, Nanjing Medical University, Nanjing, China; 4 Centre for Pharmaceutical Regulatory Sciences, University of Macau, Macao SAR, China; 5 Department of Public Health and Medicinal Administration, Faculty of Health Sciences, University of Macau, Macao SAR, China; Kaohsiung Medical University Hospital, TAIWAN

## Abstract

**Background:**

The objective of this study was to systematically analyse methodological and structural assumptions utilised in model-based health economic evaluations of systemic advanced hepatocellular carcinoma (HCC) therapies, discuss the existing challenges, and develop methodological recommendations for future models in advanced HCC.

**Methods:**

We performed literature searches using five databases (Embase, PubMed, Web of Science, Econlit, and CNKI) up to December 4, 2022. Technology appraisals from Canada, England, Australia, and the United States were also considered. Model-based full economic evaluations of systemic advanced HCC therapies in English or Chinese met the eligibility criteria. The reporting quality was assessed by using the Consolidated Health Economic Evaluation Reporting Standards 2022 checklist.

**Results:**

Of 12,863 records retrieved, 55 were eligible for inclusion. Markov model (n = 29, 53%) and partitioned survival model (n = 27, 49%) were the most commonly used modelling techniques. Most studies were based on health-state-driven structure (n = 51, 93%), followed by treatment-line-driven structure (n = 2, 4%) and combination structure (n = 1, 2%). Only three studies (5%) adopted external real-world data to extrapolate the overall survival or calibrate the extrapolation. Few studies reported the assumptions of transition probabilities. Utility modelling approaches were state-based (n = 51, 93%) and time-to-death (n = 1, 2%). Only 13 studies (24%) reported five types of model validation. Economic evaluation results of specific treatment strategies varied among studies.

**Conclusions:**

Disease modelling for health economic evaluations of systemic therapies in advanced HCC has adopted various modelling approaches and assumptions, leading to marked uncertainties in results. By proposing methodological recommendations, we suggest that future model-based studies for health economic evaluation of HCC therapies should follow good modelling practice guidelines and improve modelling methods to generate reliable health and economic evidence.

## Introduction

Hepatocellular carcinoma (HCC) raises a huge burden to societies and patients worldwide due to its high incidence, poor prognosis, and heavy costs. With approximately 906,000 diagnoses in 2020, primary liver cancer remains the 6^th^ most frequently diagnosed cancer, and there will be over annual 1,000,000 patients affected by liver cancer by 2025 [1, 2]. HCC comprises around 90% of liver cancer cases, being the major pathological type in liver cancer [2]. A variety of risk factors for HCC have been proven, such as cirrhosis, chronic hepatitis B virus or hepatitis C virus infection, type 2 diabetes, obesity, non-alcoholic steatohepatitis, and heavy alcohol intake [2–5].

HCC progress rapidly, and most patients are diagnosed in an advanced stage. Although the emerging systemic therapies such as immune checkpoint inhibitors (ICIs)-based therapies have significantly improved the clinical outcomes in advanced HCC, the medical utilisation, productivity loss, and long and poor prognosis owing to the disease have brought a substantial economic burden to HCC patients and payers [6–8]. The costs of HCC are forecasted to exceed US$500 million and JPY 607.2 billion annually in the United States (US) and Japan [9, 10]. Therefore, value evaluations integrating clinical effectiveness and costs are critical in determining cancer therapies. Health economic evaluations are needed to estimate the cost-effectiveness of HCC therapies.

However, health economic evaluations of HCC therapies face methodological challenges. Likhitsup et al. [11] conducted a focused review of cost-effectiveness and economic burden of surveillance and treatment in HCC before January 2019. Although they mainly concluded that several surveillance treatment modalities for HCC were cost-effective, they expressed concerns about the lack of descriptions of assumptions and approaches of modelling, insufficient uncertainty analysis, and improper evidence synthesis. As the decisions concerning assumptions employed and methods taken in health economic evaluations will impact the eventual decision model-based estimates, it demands more rigorous methodological reconsiderations for health economic evaluations of advanced HCC treatments [11].

Therefore, we aimed to comprehensively investigate the approaches used in model-based health economic evaluations of systemic therapies in advanced HCC, discuss the existing challenges in the adopted model approaches and assumptions, and develop methodological recommendations for future advanced HCC models.

## Methods

The procedure of this systematic literature review complied with the Preferred Reporting Items for Systematic Reviews and Meta-Analyses (PRISMA) guidelines [12]. Its protocol was registered in PROSPERO (CRD42023397657).

### Eligibility criteria

Table A in S1 Appendix presents the eligibility criteria for this review in accordance with the PICOS approach [13]. Studies were considered if they were model-based complete economic evaluations of systemic therapies for adult patients with advanced, metastatic or unresectable HCC. Studies published in English or Chinese were included. Partial or trial-based economic evaluations, conference abstracts, commentaries, editorials, reviews, animal studies, and *in vitro* studies were excluded.

### Data sources and search strategies

We performed literature searches using five databases, including Embase, PubMed, Web of Science, Econlit, and China National Knowledge Infrastructure (CNKI). In addition, relevant appraisals from four health technology assessment (HTA) agencies, including the Canadian Agency for Drugs and Technologies in Health (CADTH), the National Institute for Health and Care Excellence (NICE), the Medical Services Advisory Committee in Australia, and the Institute for Clinical and Economic Review, were also considered. The period of searches covered inception through December 4, 2022. “Hepatocellular carcinoma”, “economic”, “pharmacoeconomic”, and “cost” were used as the main search terms. Table B in S1 Appendix summarises the details of search strategies.

### Study selection

Three authors (HZ, YX, and XC) independently performed two rounds of study selection. Firstly, irrelevant results were excluded by screening retrieved titles and abstracts. Secondly, eligible studies were included by reviewing potential full texts. The references cited in included studies were also examined. In case of disagreements, two other authors (HH and COLU) were to assess and resolve their differences.

### Data extraction

One author (HZ) conducted the data extraction, with two other authors (YX and XC) verifying all the extracted information. Data elements consisted of the following aspects: first author, publication year, location, base case population, study design, treatment strategies, outcomes of economic evaluations, modelling technique, model structure, health states, progression, time horizon, cycle length, validation efforts, sensitivity analyses, clinical data source, time-to-event distributions, transition probabilities, study perspective, utility modelling approach, disutility, cost scope, and discount rates.

### Quality assessment

Two authors (HZ and YX) separately assessed the quality of peer-reviewed publications using the Consolidated Health Economic Evaluation Reporting Standards (CHEERS) 2022 checklist [14], and two other authors (HH and COLU) resolved the disagreements. Each CHEERS item was described as “reported”, “not reported”, and “not applicable”. We calculated a study-level quality score as the percentage of reported items in applicable ones. Since HTA agencies had assessed the model specifications and described the quality of models critically in technology appraisals (TAs), further description of appraisal quality was not conducted in this review.

## Results

### Search results

**Fig 1:**
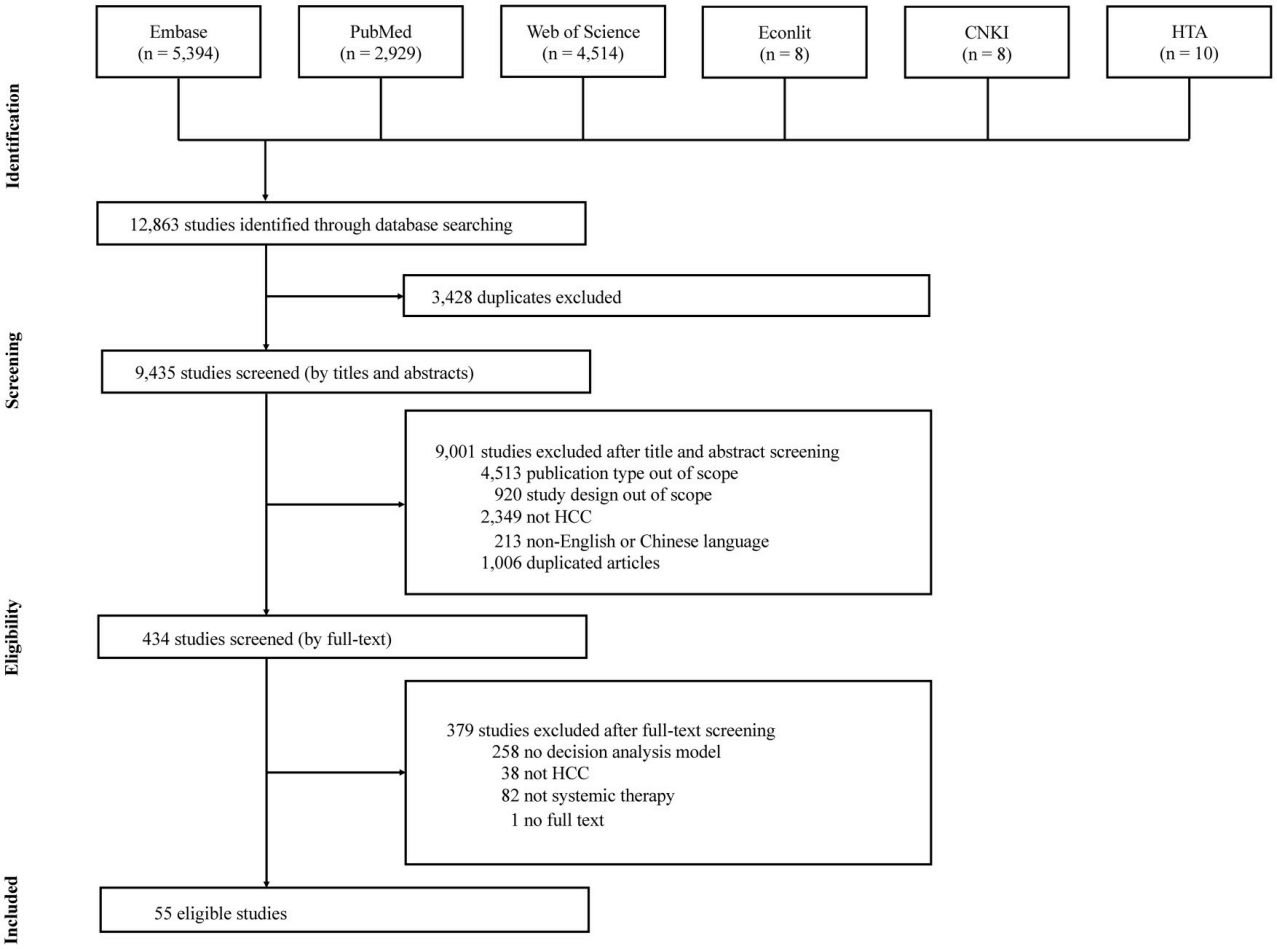
PRISMA flowchart of study selection. *CNKI, China National Knowledge Infrastructure; HCC, hepatocellular carcinoma; HTA, health technology assessment.

Searches from databases and websites of HTA agencies yielded 12,863 studies. After removing duplicates, 9,435 records were screened according to titles and abstracts, which resulted in 434 remaining to be assessed for the full text. In full-text screening, 379 studies were excluded owing to the absence of a decision analysis model or systemic therapy for HCC. Finally, 55 studies (45 publications and 10 TAs) met the eligibility criteria (Fig 1), of which 10 appraisal documents comprising company submissions and all relevant evaluations (e.g., Evidence Review Group reports) were identified from pan-Canadian Oncology Drug Review (pCODR, n = 5) and NICE (n = 5).

### Quality assessment

**Fig 2:**
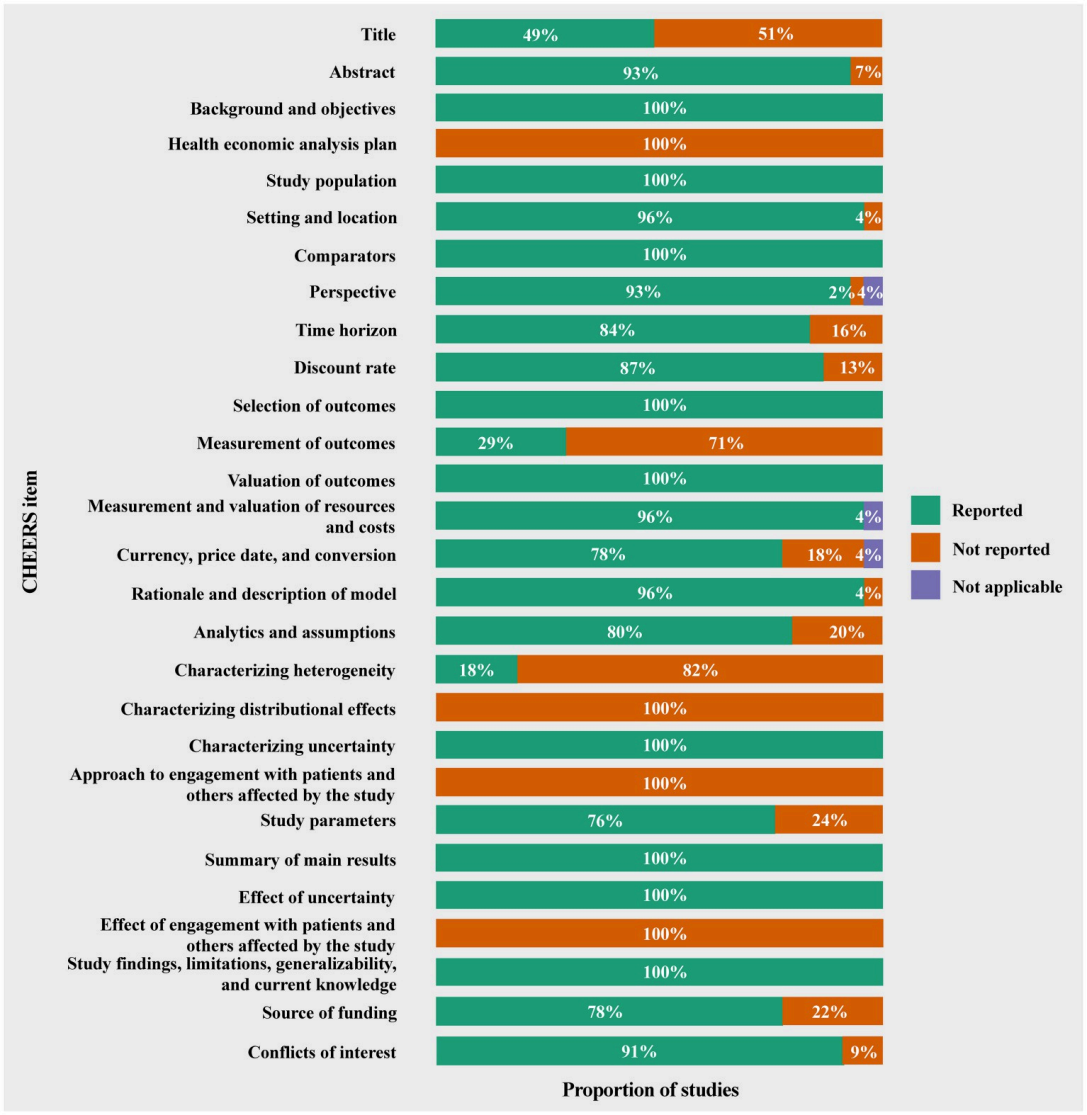
Summary of the percentage of included publications reporting each CHEERS item.

The quality of included publications was described as the study-level quality scores in Table C in S1 Appendix. The percentage of each CHEERS item correctly reported is shown in Fig 2. Reporting quality scores varied from 57% to 86%. One publication (2% of all publications), 40 publications (89%), and four publications (9%) scored < 60%, 60%-80%, and ≥ 80%, respectively (Table C in S1 Appendix). Some items were not often appropriately reported (Fig 2). Thirty-two publications (71%) did not report the effectiveness measurement decision and thirty-seven (82%) did not characterize the heterogeneity. No publication followed a health economic analysis plan, engaged with patients and others in the research design, and characterized the distributional effects in the results. No study was excluded after the quality assessment since the objective of this review was to comprehensively summarize the modelling methods of systemic therapies in advanced HCC.

### General characteristics

General characteristics of included publications and TAs were presented in Tables 1 and 2. Nineteen studies were conducted for China (35% of all studies), followed by the US (n = 16, 29%), Canada (n = 8, 15%), the United Kingdom (UK, n = 6, 11%), and Japan (n = 2, 4%). The modelled patient populations across identified studies were adults diagnosed as advanced, metastatic or unresectable HCC. Cost-utility analyses (n = 51, 93%) were the most used primary analyses, which considered quality-adjusted life-year (QALY) as the measure of health outcome. Of the remaining studies with life-year gained (LYG) serving as a health outcome, two performed cost-effectiveness analyses (4%), while the other two performed risk-benefit analyses (4%). Regarding the treatment setting, 37 studies (67%) concerned first-line therapies and 15 studies (27%) concerned second-line therapies, whereas the remaining three (5%) compared distinct sequences of therapies. The comparisons of tyrosine kinase inhibitors (TKIs) versus best supportive care (BSC, n = 16, 29%) and different TKIs-based treatments (n = 16, 29%) were the most frequently evaluated, followed by ICIs plus anti-vascular endothelial growth factor (VEGF) antibodies compared with TKIs (n = 13, 24%), ICIs against BSC/placebo (n = 3, 5%), different ICIs-based regimens (n = 2, 4%), and chemotherapy regimens compared with TKIs (n = 2, 4%). Nine studies (16%) were funded by industry, and six studies (11%) were not supported by specific funding but the authors were employed by industry.

**Table 1:** General characteristics of included full-text publications.

Study	Location	Base case population	Study design	Treatment line of focus	Interventions compared	Funding
Cabibbo et al. (2020) [28]	-	Advanced HCC	Risk-benefit analysis	Sequences	Lenvatinib-nivolumab vs. lenvatinib-pembrolizumab vs. atezolizumab plus bevacizumab-nivolumab vs. sorafenib-nivolumab vs. atezolizumab plus bevacizumab-pembrolizumab vs. lenvatinib-ramucirumab vs. lenvatinib-regorafenib vs. lenvatinib-cabozantinib vs. sorafenib-pembrolizumab vs. atezolizumab plus bevacizumab-ramucirumab vs. atezolizumab plus bevacizumab-regorafenib vs. atezolizumab plus bevacizumab-cabozantinib vs. sorafenib-cabozantinib vs. sorafenib-regorafenib vs. sorafenib-ramucirumab	Not specified, but industry employed
Cabibbo et al. (2022) [29]	-	Advanced HCC	Risk-benefit analysis	Sequences	Atezolizumab plus bevacizumab-lenvatinib vs. atezolizumab plus bevacizumab-sorafenib after 2018 vs. atezolizumab plus bevacizumab-cabozantinib vs. atezolizumab plus bevacizumab-regorafenib vs. atezolizumab plus bevacizumab-sorafenib before 2018 vs. atezolizumab plus bevacizumab-ramucirumab	Not specified, but industry employed
Cai et al. (2020) [30]	China	Untreated, nonresected advanced HCC	Cost-utility analysis	First line	Lenvatinib vs. sorafenib	Non-industry
Camma et al. (2013) [31]	Italy	Advanced HCC and intermediate HCC not eligible to or failed ablative therapies	Cost-utility analysis	First line	Full-dose sorafenib vs. BSC Dose-adjusted sorafenib vs. BSC	Not specified, but industry employed
Carr et al. (2010) [15]	US	Treatment-naïve advanced HCC	Cost-effectiveness analysis	First line	Sorafenib vs. BSC	Industry
Chiang et al. (2020) [32]	US	Advanced HCC previously treated with sorafenib	Cost-utility analysis	Second line	Pembrolizumab vs. placebo	Not specified, but industry employed
Chiang et al. (2021) [33]	US	Unresectable HCC	Cost-utility analysis	First line	Atezolizumab plus bevacizumab vs. sorafenib	Industry
Elsisi et al. (2019) [19]	Egypt	Advanced HCC	Cost-utility analysis	First line	Sorafenib vs. BSC	Not specified, but non-industry employed
Guan et al. (2022) [20]	China	Advanced HCC	Cost-utility analysis	First line	Donafenib vs. sorafenib vs. lenvatinib	Non-industry
Gupta et al. (2019) [18]	India	Advanced HCC	Cost-utility analysis	First line	Sorafenib vs. BSC	Not specified, but non-industry employed
Ho et al. (2018) [34]	Taiwan	Advanced HCC	Cost-utility analysis	First line	Sorafenib combination therapy vs. sorafenib monotherapy	Non-industry
Hou et al. (2020) [35]	China	Advanced metastatic or unresectable HCC	Cost-utility analysis	First line	Atezolizumab plus bevacizumab vs. sorafenib	Not specified, but non-industry employed
Ikeda et al. (2021) [36]	Japan	Unresectable HCC	Cost-utility analysis	First line	Lenvatinib vs. sorafenib	Industry
Kim et al. (2020) [37]	Canada	Unresectable HCC	Cost-utility analysis	First line	Lenvatinib vs. sorafenib	Non-industry
Kobayashi et al. (2019) [38]	Japan	Intermediate and advanced unresectable HCC	Cost-utility analysis	First line	Lenvatinib vs. sorafenib	Not specified, but industry employed
Li et al. (2021) [39]	China	Advanced HCC with portal vein invasion	Cost-utility analysis	First line	HAIC plus sorafenib vs. sorafenib	Non-industry
Li et al. (2022) [24]	China	Advanced or unresectable HCC	Cost-utility analysis	First line	Sintilimab plus bevacizumab biosimilar vs. sorafenib Atezolizumab plus bevacizumab vs. sorafenib	Not specified, but non-industry employed
Li et al. (2022) [40]	US	Advanced or unresectable HCC	Cost-utility analysis	First line	Atezolizumab plus bevacizumab vs. nivolumab	Non-industry
Liao et al. (2019) [41]	US, UK and China	Sorafenib‐resistant HCC	Cost-utility analysis	Second line	Cabozantinib vs. BSC	Non-industry
Liu et al. (2022) [42]	China and US	Locally advanced, metastatic or unresectable HCC	Cost-utility analysis	First line	Atezolizumab plus bevacizumab vs. sorafenib	Non-industry
Meng et al. (2021) [43]	China	Advanced HCC previously treated with sorafenib	Cost-utility analysis	Second line	Pembrolizumab vs. placebo	Non-industry
Meng et al. (2022) [23]	China	Unresectable or metastatic HCC	Cost-utility analysis	First line	Donafenib vs. sorafenib	Not specified, but non-industry employed
Meng et al. (2022) [21]	China	Unresectable or metastatic HCC	Cost-utility analysis	First line	Donafenib vs. lenvatinib	Not specified, but non-industry employed
Meyers et al. (2021) [44]	Canada	Untreated advanced or unresectable HCC	Cost-utility analysis	First line	Lenvatinib vs. sorafenib	Industry
Muszbek et al. (2008) [16]	Canada	Treatment-naïve advanced HCC	Cost-effectiveness analysis	First line	Sorafenib vs. BSC	Industry
Parikh et al. (2017) [45]	US	Advanced HCC previously treated with sorafenib	Cost-utility analysis	Second line	Regorafenib vs. BSC	Not specified, but industry employed
Peng et al. (2022) [25]	China	Unresectable HCC	Cost-utility analysis	First line	Sintilimab plus bevacizumab biosimilar vs. sorafenib	Non-industry
Qin et al. (2018) [46]	China	Advanced or metastatic HCC	Cost-utility analysis	First line	FOLFOX4 vs. sorafenib	Industry
Saiyed et al. (2020) [47]	Australia	Treatment-naïve advanced HCC	Cost-utility analysis	First line	Lenvatinib vs. sorafenib	Not specified, but non-industry employed
Sangmala et al. (2018) [48]	Thailand	Advanced HCC	Cost-utility analysis	First line	Sorafenib vs. palliative care	Not specified, but non-industry employed
Sherrow et al. (2020) [49]	US	Advanced HCC	Cost-utility analysis	Sequences	Sorafenib-regorafenib vs. sorafenib-cabozantinib vs. sorafenib-pembrolizumab vs. sorafenib-nivolumab vs. lenvatinib-regorafenib vs. lenvatinib-cabozantinib vs. lenvatinib-pembrolizumab vs. lenvatinib-nivolumab	Industry
Shi et al. (2021) [50]	China	Advanced HCC previously treated with sorafenib or systemic chemotherapy	Cost-utility analysis	Second line	Two-week regimen of camrelizumab vs. three-week regimen of camrelizumab	Not specified, but non-industry employed
Shlomai et al. (2018) [51]	US	Advanced HCC previously treated with sorafenib	Cost-utility analysis	Second line	Regorafenib vs. BSC	Not specified, but non-industry employed
Shlomai et al. (2019) [52]	US	Advanced HCC	Cost-utility analysis	Second line	Cabozantinib vs. BSC	Not specified, but non-industry employed
Sieg et al. (2020) [53]	Germany and US	Advanced HCC received prior sorafenib	Cost-utility analysis	Second line	Cabozantinib vs. BSC	Not specified, but non-industry employed
Soto-Perez-de-Celis et al. (2019) [54]	US	Advanced HCC received prior sorafenib	Cost-utility analysis	Second line	Cabozantinib vs. BSC	Not specified, but non-industry employed
Su et al. (2021) [55]	US	Unresectable HCC	Cost-utility analysis	First line	Atezolizumab plus bevacizumab vs. sorafenib	Non-industry
Wen et al. (2021) [56]	China and US	Unresectable HCC	Cost-utility analysis	First line	Atezolizumab plus bevacizumab vs. sorafenib	Non-industry
Zhang et al. (2015) [17]	China	Advanced HCC	Cost-utility analysis	First line	Sorafenib vs. BSC	Not specified, but non-industry employed
Zhang et al. (2016) [57]	China	Advanced HCC	Cost-utility analysis	First line	FOLFOX4 vs. sorafenib	Not specified, but non-industry employed
Zhang et al. (2021) [58]	US	Locally advanced metastatic or unresectable HCC	Cost-utility analysis	First line	Atezolizumab plus bevacizumab vs. sorafenib	Non-industry
Zhao et al. (2022) [22]	China	Unresectable HCC	Cost-utility analysis	First line	Sorafenib vs. lenvatinib vs. donafenib vs. sintilimab plus bevacizumab biosimilar vs. atezolizumab plus bevacizumab	Non-industry
Zheng et al. (2020) [59]	US	Advanced HCC received prior sorafenib with α-fetoprotein concentrations of at least 400 ng/ml	Cost-utility analysis	Second line	Ramucirumab vs. placebo	Non-industry
Zhou et al. (2022) [26]	China	Unresectable or metastatic HCC	Cost-utility analysis	First line	Sintilimab plus bevacizumab biosimilar vs. sorafenib	Industry
Zhou et al. (2022) [27]	China	Unresectable or metastatic HCC	Cost-utility analysis	First line	Sintilimab plus bevacizumab biosimilar vs. lenvatinib	Industry

Abbreviations: BSC, best supportive care; HAIC, hepatic arterial infusion of chemotherapy; HCC, hepatocellular carcinoma; UK, United Kingdom; US, United States.

**Table 2:** General characteristics of included technology appraisals.

TA	Location	Base case population	Study design	Treatment line of focus	Interventions compared	Funding
NICE TA 474 (2017) [60]	UK	Advanced HCC when surgical or locoregional therapies had failed or were not suitable	Cost-utility analysis	First line	Sorafenib vs. BSC	Non-industry
NICE TA 551 (2018) [61]	UK	Untreated, advanced, unresectable HCC	Cost-utility analysis	First line	Lenvatinib vs. sorafenib	Non-industry
NICE TA 555 (2019) [62]	UK	Advanced unresectable HCC previously treated with sorafenib	Cost-utility analysis	Second line	Regorafenib vs. BSC	Non-industry
NICE TA 666 (2020) [63]	UK	Advanced or unresectable HCC	Cost-utility analysis	First line	Atezolizumab plus bevacizumab vs. sorafenib Atezolizumab plus bevacizumab vs. lenvatinib	Non-industry
NICE TA 849 (2022) [64]	UK	Advanced HCC previously treated with sorafenib	Cost-utility analysis	Second line	Cabozantinib vs. regorafenib	Non-industry
pCODR 10119 (2018) [65]	Canada	Unresectable HCC following treatment with sorafenib	Cost-utility analysis	Second line	Regorafenib vs. BSC	Non-industry
pCODR 10134 (2018) [66]	Canada	Sorafenib-refractory or intolerant advanced (not amenable to curative therapy or local therapeutic measures) or metastatic HCC	Cost-utility analysis	Second line	Nivolumab vs. BSC	Non-industry
pCODR 10175 (2019) [67]	Canada	Advanced, unresectable HCC	Cost-utility analysis	First line	Lenvatinib vs. sorafenib	Non-industry
pCODR 10186 (2020) [68]	Canada	Advanced HCC previously treated with sorafenib	Cost-utility analysis	Second line and third line	Cabozantinib vs. BSC Cabozantinib vs. regorafenib	Non-industry
pCODR 10217 (2020) [69]	Canada	Unresectable or metastatic HCC	Cost-utility analysis	First line	Atezolizumab plus bevacizumab vs. sorafenib Atezolizumab plus bevacizumab vs. lenvatinib	Non-industry

Abbreviations: BSC, best supportive care; HCC, hepatocellular carcinoma; NICE, National Institute for Health and Care Excellence; pCODR, pan-Canadian Oncology Drug Review; TA, technology appraisal; UK, United Kingdom.

### Economic evaluation results

Economic evaluation results of the similar or same treatment settings for advanced HCC varied among studies (Tables D and E in S1 Appendix). For instance, first-line sorafenib compared with BSC resulted in cost-effectiveness from two studies conducted for the US (ICER US$62,473/LY gained, willingness-to-pay threshold of US$100,000/LY, 2007 values) and Canada (ICER Can$75,759/LY gained, willingness-to-pay threshold of Can$100,000/LY, 2007 values) [15, 16], while one Chinese study, one Indian study, and one Egyptian study reported that sorafenib was not cost-effective with ICERs of US$101,399.11/QALY gained (willingness-to-pay threshold of US$20,301/QALY, 2012 values), US$7,861/QALY gained (willingness-to-pay threshold of 1–3 times the GDP per capita for India, 2017 values), and US$286,776/QALY gained (willingness-to-pay threshold of US$41,372/QALY, 2017 values), respectively [17–19]. Of the Chinese studies including first-line treatment with donafenib, all but one showed improved cost-effectiveness in donafenib in comparison with sorafenib and lenvatinib [20–22], whereas the other study considered that donafenib was not a cost-effective strategy compared to sorafenib with an ICER of US$41,081.52/QALY gained (willingness-to-pay threshold of US$10,499.74–31,499.23/QALY, 2020 values) [23]. Regarding initial treatment with sintilimab plus bevacizumab biosimilar, one Chinese analysis revealed that it lacked cost-effectiveness when compared with sorafenib (ICER US$51,877.36/QALY gained), lenvatinib (ICER US$56,890.35/QALY gained), and donafenib (ICER US$66,487.88/QALY gained, willingness-to-pay threshold of US$33,521/QALY, 2020 values) [22], although the other four analyses showed a cost-effective profile of sintilimab plus bevacizumab biosimilar in China [24–27]. For other treatment strategies, significant differences in results were also observed among studies, for which the reasons were probably that different modelling approaches and assumptions were adopted.

### Decision model characteristics

Table 3 and Tables F and G in S1 Appendix summarised the main methodological characteristics of included decision models consisting of modelling technique and structure, time horizon, cycle length, discounting, clinical effectiveness inputs, utility and cost inputs, sensitivity analyses, and validation efforts.

**Table 3:** Summary of included decision models.

Study	Modelling technique	Model structure	Health states	Progression	Time horizon	Cycle length	Discount rate	Validation efforts	Sensitivity analyses
Cabibbo et al. (2020) [28]	Markov model	Treatment lines	1^st^ line treatment, 2^nd^ line treatment, death	1^st^ line treatment-1^st^ line treatment 1^st^ line treatment-2^nd^ line treatment 2^nd^ line treatment-2^nd^ line treatment 2^nd^ line treatment-death	Lifetime	1 month	-	-	1-way SA, 2-way SA, PSA
Cabibbo et al. (2022) [29]	Markov model	Treatment lines	1^st^ line treatment, 2^nd^ line treatment, death	1^st^ line treatment-1^st^ line treatment 1^st^ line treatment-2^nd^ line treatment 2^nd^ line treatment-2^nd^ line treatment 2^nd^ line treatment-death	Lifetime	1 month	-	-	2-way SA
Cai et al. (2020) [30]	Markov model	Health states	PFS, PD, death	PFS-PFS PFS-PD PD-PD PD-death	10 years	1 month	3%	-	1-way SA, PSA
Camma et al. (2013) [31]	Markov model	Health states	BCLC B HCC, BCLC C HCC, death	BCLC B HCC-death BCLC C HCC-death	5 years	-	3%	Face validity, internal	1-way SA, PSA
Carr et al. (2010) [15]	Markov model	Health states	1^st^ line treatment-no progression, 1^st^ line treatment continued-post progression, BSC-post progression, death	Sorafenib: 1^st^ line no progression-1^st^ line no progression 1^st^ line no progression-1^st^ line continued post progression 1^st^ line no progression-BSC 1^st^ line no progression-death 1^st^ line continued post progression-1^st^ line continued post progression 1^st^ line continued post progression-BSC 1^st^ line continued post progression-death BSC-BSC BSC-death BSC: 1^st^ line no progression-1^st^ line no progression 1^st^ line no progression-BSC post-progression 1^st^ line no progression-death BSC post-progression-BSC post-progression BSC post-progression-death	14 years	1 month	3%	Face validity	1-way SA, PSA, scenario analyses
Chiang et al. (2020) [32]	Markov model	Health states	PFS, PD, death	PFS-PFS PFS-death PFS-PD PD-PD PD-death	3 years	3 weeks	3%	-	1-way SA, 2-way SA, PSA, scenario analyses, subgroup analyses
Chiang et al. (2021) [33]	Markov model	Health states	PFS, PD, death	PFS-PFS PFS-death PFS-PD PD-PD PD-death	5 years	3 weeks	3%	-	1-way SA, 2-way SA, PSA, scenario analyses, subgroup analyses
Elsisi et al. (2019) [19]	Markov model	Health states	PFS, PD, death	PFS-PFS PFS-death PFS-PD PD-PD PD-death	4 years	1 month	3.5%	Face validity	1-way SA, PSA
Guan et al. (2022) [20]	Partitioned survival model	Health states	PFS, PD, death	PFS-PFS PFS-death PFS-PD PD-PD PD-death	Lifetime	1 week	5%	-	1-way SA, PSA, scenario analyses
Gupta et al. (2019) [18]	Markov model	Health states	PFS, PD, death	PFS-PFS PFS-death PFS-PD PD-PD PD-death	Lifetime	1 month	3%	-	PSA, scenario analyses
Ho et al. (2018) [34]	Markov model	Health states	PFS, PD, death	PFS-PFS PFS-death PFS-PD PD-PD PD-death	5 years	1 month	3%	-	1-way SA, PSA
Hou et al. (2020) [35]	Partitioned survival model	Health states	PFS, PD, death	PFS-PFS PFS-death PFS-PD PD-PD PD-death	-	1 week	5%	-	1-way SA, PSA, subgroup analyses
Ikeda et al. (2021) [36]	Partitioned survival model	Health states	PFS, PD, death	PFS-PFS PFS-death PFS-PD PD-PD PD-death	20 years	4 weeks	2%	-	1-way SA, PSA, scenario analyses
Kim et al. (2020) [37]	Markov model	Health states	PFS, PD, death	PFS-PFS PFS-death PFS-PD PD-PD PD-death	5 years	1 month	1.5%	-	PSA, subgroup analyses
Kobayashi et al. (2019) [38]	Partitioned survival model	Health states	PFS, PD, death	PFS-PFS PFS-death PFS-PD PD-PD PD-death	Lifetime	4 weeks	2%	-	DSA, PSA, scenario analyses
Li et al. (2021) [39]	Markov model	Health states	PFS, RFS, PD, death	PFS-PFS PFS-RFS PFS-PD PFS-death RFS-RFS RFS-PD RFS-death PD-PD PD-death	8 years	3 weeks	3%	-	1-way SA, PSA
Li et al. (2022) [24]	Partitioned survival model	Health states	PFS, PD, death	PFS-PFS PFS-death PFS-PD PD-PD PD-death	15 years	1 month	5%	-	1-way SA, PSA, scenario analyses
Li et al. (2022) [40]	Partitioned survival model	Health states	PFS, PD, death	PFS-PFS PFS-death PFS-PD PD-PD PD-death	10 years	1 month	3%	-	1-way SA, PSA, subgroup analyses
Liao et al. (2019) [41]	Markov model	Health states	PFS, PD, death	PFS-death PFS-PD PD-death	10 years	1 month	3%	Face validity, calibration	1-way SA, PSA
Liu et al. (2022) [42]	Markov model and partitioned survival model	Health states	PFS, PD, death	PFS-PFS PFS-death PFS-PD PD-PD PD-death	10 years	1 month	China: 5% US: 3%	-	1-way SA, PSA, scenario analyses
Meng et al. (2021) [43]	Markov model and partitioned survival model	Health states	PFS, PD, death	PFS-PFS PFS-death PFS-PD PD-PD PD-death	Lifetime	3 weeks	5%	-	1-way SA, PSA, scenario analyses
Meng et al. (2022) [23]	Partitioned survival model	Health states	PFS, PD, death	PFS-PFS PFS-death PFS-PD PD-PD PD-death	Lifetime	4 weeks	5%	-	1-way SA, PSA, scenario analyses
Meng et al. (2022) [21]	Partitioned survival model	Health states	PFS, PD, death	PFS-PFS PFS-death PFS-PD PD-PD PD-death	Lifetime	4 weeks	5%	-	1-way SA, PSA, scenario analyses
Meyers et al. (2021) [44]	Partitioned survival model	Health states	PFS, PD, death	PFS-PFS PFS-death PFS-PD PD-PD PD-death	10 years	4 weeks	1.5%	Face validity	1-way SA, PSA, scenario analyses
Muszbek et al. (2008) [16]	Markov model	Health states	1^st^ line treatment-no progression, 1^st^ line treatment continued-post progression, BSC-post progression, death	Sorafenib: 1^st^ line no progression-1^st^ line no progression 1^st^ line no progression-1^st^ line continued post progression 1^st^ line no progression-BSC 1^st^ line no progression-death 1^st^ line continued post progression-1^st^ line continued post progression 1^st^ line continued post progression-BSC 1^st^ line continued post progression-death BSC-BSC BSC-death BSC: 1^st^ line no progression-1^st^ line no progression 1^st^ line no progression-BSC post-progression 1^st^ line no progression-death BSC post-progression-BSC post-progression BSC post-progression-death	14 years	1 month	5%	Face validity	1-way SA, PSA, scenario analyses
NICE TA 474 (2017) [60]	Markov model	Health states	1^st^ line treatment-no progression, 1^st^ line treatment continued-post progression, BSC-post progression, death	Sorafenib: 1^st^ line no progression-1^st^ line no progression 1^st^ line no progression-1^st^ line continued post progression 1^st^ line no progression-BSC 1^st^ line no progression-death 1^st^ line continued post progression-1^st^ line continued post progression 1^st^ line continued post progression-BSC 1^st^ line continued post progression-death BSC-BSC BSC-death BSC: 1^st^ line no progression-1^st^ line no progression 1^st^ line no progression-BSC post-progression 1^st^ line no progression-death BSC post-progression-BSC post-progression BSC post-progression-death	14 years	1 month	3.5%	Face validity, internal, external	1-way SA, 2-way SA, PSA, scenario analyses, subgroup analyses
NICE TA 551 (2018) [61]	Partitioned survival model	Health states	PFS, PD, death	-	20 years	1 month	3.5%	-	Scenario analyses
NICE TA 555 (2019) [62]	Partitioned survival model	Health states	PFS, PD, death	-	15 years	-	-	-	1-way SA, PSA, scenario analyses
NICE TA 666 (2020) [63]	Partitioned survival model	Health states	PFS, PD, death	PFS-PFS PFS-death PFS-PD PD-PD PD-death	20 years	1 week	3.5%	Face validity, internal, cross-validation, external	1-way SA, PSA, scenario analyses
NICE TA 849 (2022) [64]	Partitioned survival model	Health states	PFS, PD, death	PFS-PFS PFS-death PFS-PD PD-PD PD-death	15 years	4 weeks	3.5%	Face validity, internal, cross-validation	1-way SA, PSA, scenario analyses
Parikh et al. (2017) [45]	Markov model	Health states	PFS, PD, death	PFS-death PFS-PD PD-death	-	1 week	3%	-	1-way SA, 2-way SA, PSA
pCODR 10119 (2018) [65]	Partitioned survival model	Health states	PFS, PD, death	-	3 years	-	-	-	1-way SA, scenario analyses
pCODR 10134 (2018) [66]	Partitioned survival model	Health states	PFS, PD, death	PFS-PFS PFS-death PFS-PD PD-PD PD-death	3 years	1 week	-	-	1-way SA, PSA, scenario analyses
pCODR 10175 (2019) [67]	Partitioned survival model	Health states	PFS, PD, death	PFS-PFS PFS-death PFS-PD PD-PD PD-death	10 years	-	-	-	1-way SA, PSA, scenario analyses
pCODR 10186 (2020) [68]	Partitioned survival model	Health states	PFS, PD, death	PFS-PFS PFS-death PFS-PD PD-PD PD-death	5 years	1 month	-	-	PSA, scenario analyses
pCODR 10217 (2020) [69]	Partitioned survival model	Health states	PFS, PD, death	-	10 years	-	-	-	-
Peng et al. (2022) [25]	Markov model	Health states	PFS, PD, death	PFS-PFS PFS-death PFS-PD PD-PD PD-death	Lifetime	3 weeks	3%	-	1-way SA, PSA
Qin et al. (2018) [46]	Markov model	Health states	PFS, PD, death	PFS-PFS PFS-death PFS-PD PD-PD PD-death	Lifetime	1 month	5%	Face validity	1-way SA, PSA, scenario analyses
Saiyed et al. (2020) [47]	Partitioned survival model	Health states	PFS, PD, death	PFS-PFS PFS-death PFS-PD PD-PD PD-death	10 years	1 month	5%	External, cross-validation	1-way SA, PSA, scenario analyses
Sangmala et al. (2018) [48]	Markov model	Health states	1^st^ line treatment-no progression, 1^st^ line treatment continued-post progression, palliative care-post progression, death	Sorafenib: 1^st^ line no progression-1^st^ line no progression 1^st^ line no progression-1^st^ line continued post progression 1^st^ line no progression-palliative care 1^st^ line no progression-death 1^st^ line continued post progression-1^st^ line continued post progression 1^st^ line continued post progression-palliative care 1^st^ line continued post progression-death Palliative care-palliative care Palliative care-death Palliative care: 1^st^ line no progression-1^st^ line no progression 1^st^ line no progression-palliative care post- progression 1^st^ line no progression-death Palliative care post- progression-palliative care post-progression Palliative care post- progression-death	Lifetime	1 month	3%	-	1-way SA, PSA
Sherrow et al. (2020) [49]	Markov model	Health states + treatment lines	1^st^ line: 1^st^ line treatment, 1^st^ line treatment with toxicity, 1^st^ line discontinuation due to toxicity, 1^st^ line progression, death 2^nd^ line: 2^nd^ line treatment, 2^nd^ line treatment with toxicity, 2^nd^ line discontinuation due to toxicity, 2^nd^ line progression, death	1^st^ line treatment-1^st^ line treatment 1^st^ line treatment-1^st^ line w/toxicity 1^st^ line treatment-discontinue 1^st^ line 1^st^ line treatment-1^st^ line progression 1^st^ line treatment-death 1^st^ line w/toxicity-1^st^ line treatment 1^st^ line w/toxicity-1^st^ line w/toxicity 1^st^ line w/toxicity-discontinue 1^st^ line 1^st^ line w/toxicity-1^st^ line progression 1^st^ line w/toxicity-death Discontinue 1^st^ line-2^nd^ line treatment Discontinue 1^st^ line-2^nd^ line w/toxicity Discontinue 1^st^ line-discontinue 2^nd^ line Discontinue 1^st^ line-death 1^st^ line progression-2^nd^ line treatment 1^st^ line progression-2^nd^ line w/toxicity 1^st^ line progression-discontinue 2^nd^ line 1^st^ line progression-death 2^nd^ line treatment-2^nd^ line treatment 2^nd^ line treatment-2^nd^ line w/toxicity 2^nd^ line treatment-discontinue 2^nd^ line 2^nd^ line treatment-2^nd^ line progression 2^nd^ line treatment-death 2^nd^ line w/toxicity-2^nd^ line treatment 2^nd^ line w/toxicity-2^nd^ line w/toxicity 2^nd^ line w/toxicity-discontinue 2^nd^ line 2^nd^ line w/toxicity-2^nd^ line progression 2^nd^ line w/toxicity-death Discontinue 2^nd^ line-discontinue 2^nd^ line Discontinue 2^nd^ line-2^nd^ line progression Discontinue 2^nd^ line-death 2^nd^ line progression-2^nd^ line progression 2^nd^ line progression-death	-	1 month	-	Face validity	PSA
Shi et al. (2021) [50]	Partitioned survival model	Health states	PFS, PD, death	PFS-PFS PFS-death PFS-PD PD-PD PD-death	Lifetime	Two-week regimen: 2 weeks Three-week regimen: 3 weeks	5%	-	1-way SA, PSA
Shlomai et al. (2018) [51]	Markov model	Health states	PFS without AE, PFS with AE, PD, death	PFS without AE-PFS without AE PFS without AE-PFS with AE PFS without AE-PD PFS without AE-death PFS with AE-PD PFS with AE-death PD-PD PD-death	-	4 weeks	3%	-	1-way SA, PSA
Shlomai et al. (2019) [52]	Markov model	Health states	PFS, PD, death	PFS-PD PFS-death PD-death	5 years	4 weeks	3%	-	1-way SA, PSA
Sieg et al. (2020) [53]	Markov model	Health states	PFS, PD, death	PFS-PFS PFS-death PFS-PD PD-PD PD-death	7 years	1 month	3%	-	1-way SA, PSA, scenario analyses
Soto-Perez-de-Celis et al. (2019) [54]	-	Health states	PFS, PD, death	PFS-death PFS-PD PD-death	-	-	-	-	1-way SA
Su et al. (2021) [55]	Partitioned survival model	Health states	PFS, PD, death	PFS-PFS PFS-death PFS-PD PD-PD PD-death	-	1 week	3%	-	1-way SA, PSA, subgroup analyses
Wen et al. (2021) [56]	Markov model	Health states	PFS, PD, death	PFS-PFS PFS-death PFS-PD PD-PD PD-death	10 years	1 month	3%	Calibration	1-way SA, PSA
Zhang et al. (2015) [17]	Markov model	Health states	PFS, PD, death	PFS-PFS PFS-death PFS-PD PD-PD PD-death	-	1 month	3%	-	1-way SA, subgroup analyses
Zhang et al. (2016) [57]	Markov model	Health states	PFS, PD, death	PFS-PFS PFS-death PFS-PD PD-PD PD-death	10 years	1 month	-	-	1-way SA, PSA
Zhang et al. (2021) [58]	Partitioned survival model	-	-	-	6 years	1 month	3%	-	1-way SA, PSA, subgroup analyses
Zhao et al. (2022) [22]	Partitioned survival model	Health states	PFS, PD, death	PFS-PFS PFS-death PFS-PD PD-PD PD-death	10 years	1 month	5%	-	1-way SA, PSA, scenario analyses
Zheng et al. (2020) [59]	Markov model	Health states	PFS, PD, death	PFS-PFS PFS-death PFS-PD PD-PD PD-death	10 years	1 month	3%	-	1-way SA, PSA, scenario analyses
Zhou et al. (2022) [26]	Partitioned survival model	Health states	PFS, PD, death	PFS-PFS PFS-death PFS-PD PD-PD PD-death	Lifetime	3 weeks	5%	-	1-way SA, PSA, scenario analyses
Zhou et al. (2022) [27]	Partitioned survival model	Health states	PFS, PD, death	PFS-PFS PFS-death PFS-PD PD-PD PD-death	Lifetime	3 weeks	5%	Face validity	1-way SA, PSA, scenario analyses

Abbreviations: BCLC, Barcelona Clinic Liver Cancer; BSC, best supportive care; HCC, hepatocellular carcinoma; NICE, National Institute for Health and Care Excellence; pCODR, pan-Canadian Oncology Drug Review; PD, progressive disease; PFS, progression-free survival; PSA, probabilistic sensitivity analysis; RFS, recurrence-free survival; SA, sensitivity analysis; TA, technology appraisal.

#### Summary of included decision models

Table 3 provided an overview of the general characteristics of included decision models. Markov model (n = 29, 53%) and partitioned survival model (n = 27, 49%) were the most commonly used modelling techniques for systemic therapies in advanced HCC. Two studies (4%) investigated both approaches to explore structural uncertainty [42, 43]. One study (2%) from the US did not detail the technique used [54].

**Fig 3:**
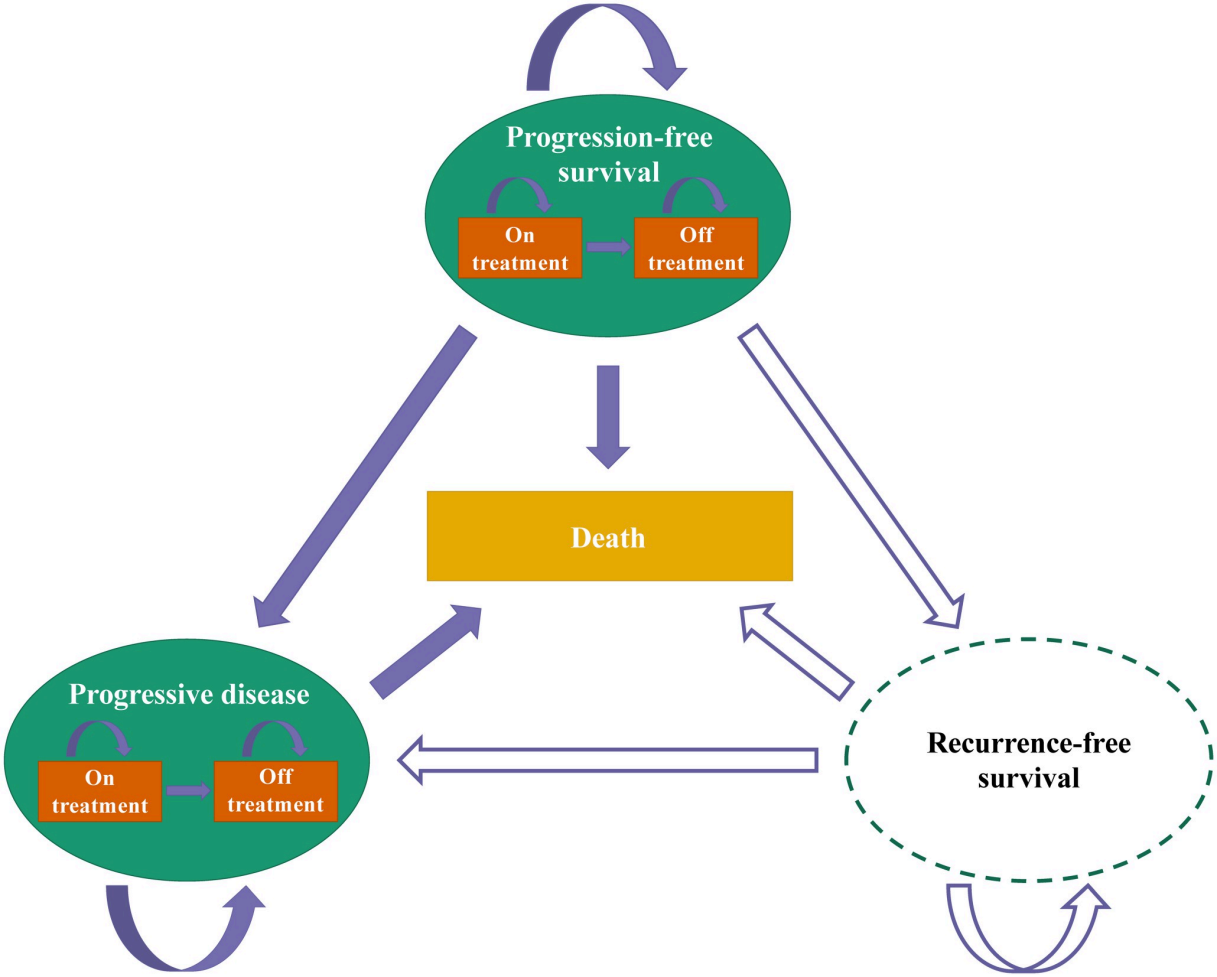
The health-state-driven structure of health economic advanced HCC models.

Concerning model structure, most studies were based on a core structure of advanced HCC defined by a disease pathway of traditional three health states: progression-free survival (PFS), progressive disease (PD), and death (n = 51, 93%). Of these models, four divided the PD state into continuous first-line treatment and off-first-line treatment after progression [15, 16, 48, 60], one model added a state in PFS to reflect intolerance (adverse events) [51], one model involved an additional recurrence-free survival (RFS) state [39], and one merged PFS and PD into one disease state [31]. The disease progression in the health-state-driven model structure was that (Fig 3), in general, advanced HCC patients receiving systemic therapy started in the PFS state, following which they could stop treatment due to intolerance (adverse events). Before transitioning to PD, patients could experience an additional state of RFS. Once in the PD state, patients could either continue or stop previous treatment. Also, the transition to death could happen from any health state in the model.

**Fig 4:**
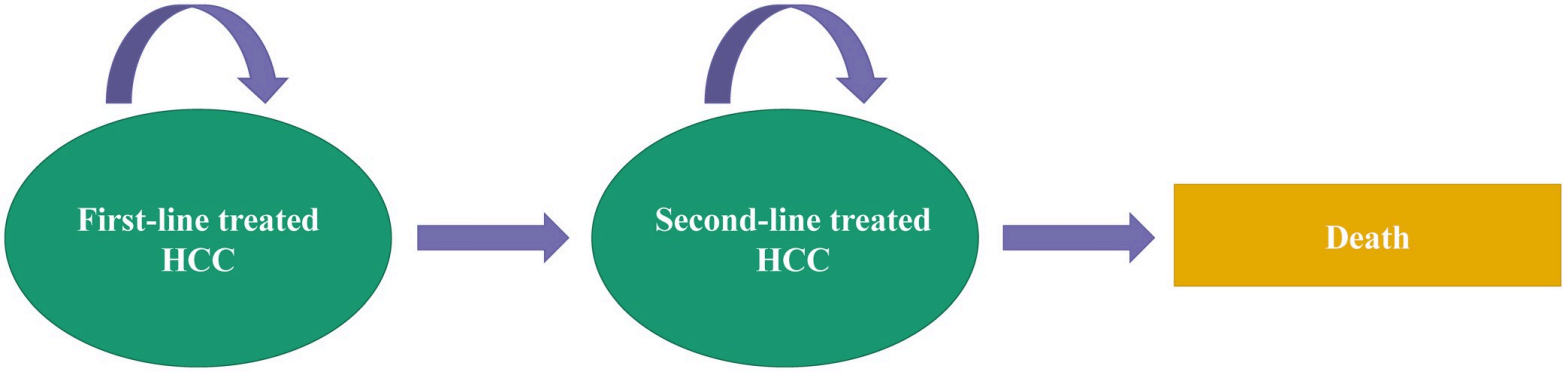
The treatment-line-driven structure of health economic advanced HCC models.

**Fig 5:**
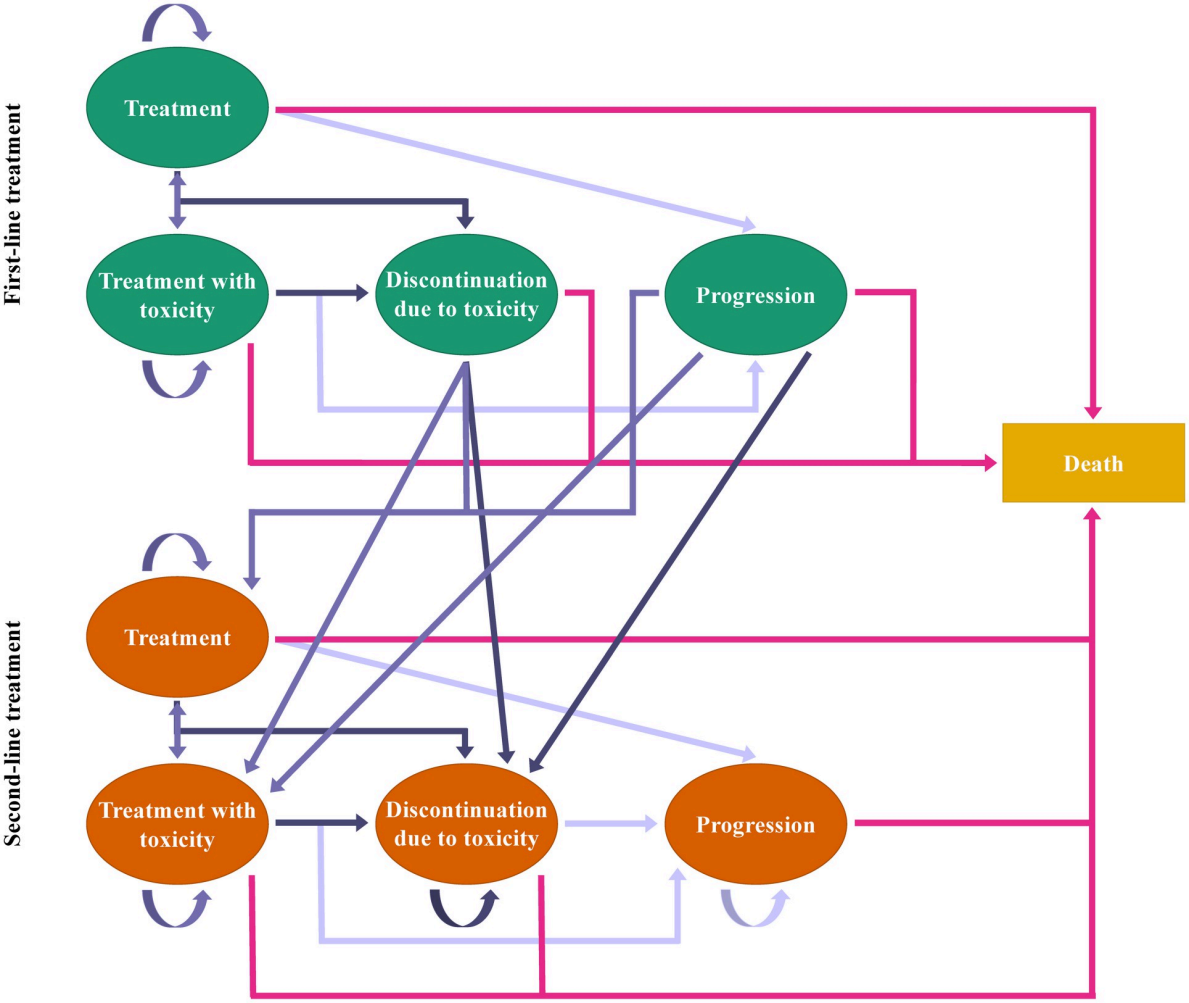
The combination structure of health economic advanced HCC models.

Two studies (4%) employed a treatment-line-driven structure in which patients could progress from receiving first-line treatment to second-line treatment and later switch to death (Fig 4) [28, 29]. One study (2%) applied a combination structure of health-state and treatment-line-driven ones, allowing nine health states for two lines of treatment [49]. Within the model, patients began by receiving first-line treatment, and they could progress to any state of the first-line treatment regardless of whether toxicity occurred or not. After first-line discontinuation owing to toxicity or progression, patients could move to the second-line treatment. There was no subsequent-line treatment if patients experienced second intolerance or progression. Similarly, death could occur at any point in the model (Fig 5).

Concerning the time horizons of included studies, varying between 3 years and lifetime, 4 studies (7%), 9 studies (16%), 21 studies (38%), and 14 studies (25%) utilized the time horizons of < 5 years, 5–10 years, 10–20 years (including 20 years), and lifetime, respectively. Seven studies (13%) did not report a time horizon. It was noteworthy that 5 years [37], 6 years [58], 7 years [53], and 8 years [39] (each n = 1), 10 years (n = 8) [22, 30, 40, 44, 47, 56, 57, 69], 14 years (n = 3) [15, 16, 60], 15 years (n = 1) [64], and 20 years (n = 3) [36, 61, 63], were assumed to equal a horizon of lifetime by researchers. Cycle lengths of 1 month (n = 27, 49%), 3 weeks (n = 8, 15%), 4 weeks (n = 8, 15%), 1 week (n = 6, 11%), and 2 weeks (n = 1, 2%), were adopted for included models. No cycle length was identified for six studies (11%). Discount rates of 1.5%, 2%, 3%, 3.5%, and 5% for health effects and costs were reported in this review.

#### Clinical effectiveness inputs

Table F in S1 Appendix summarized the clinical effectiveness inputs of identified models. Thirty-four studies (62%) used aggregate data from randomized controlled trials (RCTs) as evidence sources for clinical effectiveness, followed by individual patient data (IPD) from RCTs (n = 13, 24%), IPD from real-world data (RWD, n = 4, 7%) [19, 31, 34, 48], combinations of IPD and aggregate data from RCTs (n = 3, 5%) [27, 64, 68], and combination of IPD from RWD and aggregate data from RCT (n = 1, 2%) [17]. Of these studies, eight (15%) applied network meta-analyses to indirectly compare the key treatment efficacy using RCT aggregate data [20–22, 24, 40], RCT IPD [63, 69], and combination of RCT IPD and aggregate data [27].

Thirty-nine studies (71%) extrapolated or modelled base-case time-to-event data using parametric distributions. For PFS extrapolation, the lognormal distribution was the most commonly adopted (n = 21, 38%), followed by the Weibull distribution (n = 11, 20%), log-logistic distribution (n = 6, 11%), generalised gamma [61, 66] and Royston-Parmar spline models [35, 55] (each n = 2, 4%), and an exponential function (n = 1, 2%) [42]. Similarly, parametric extrapolations for overall survival (OS) were identified as follows: lognormal (n = 19, 35%), log-logistic (n = 14, 25%), Weibull (n = 13, 24%), generalised gamma [68], and exponential [50] (each n = 1, 2%) distributions. Moreover, in seven studies (13%), the appropriate fitting distributions were selected for survival curves of one of the treatment arms, and then the extrapolated survival curves of other arms were adjusted on the basis of HRs derived from comparisons between treatment arms [20–22, 24, 25, 27, 40]. Of these studies, most reported the treatment efficacy by conducting network meta-analyses [20–22, 24, 27, 40].

Additionally, only one study used external RWD to extrapolate the OS [33]. In Chiang et al., survival curves were parametric in base-case analysis; however, the Surveillance, Epidemiology, and End Results (SEER) data were utilized to extrapolate the OS after 17 months in the atezolizumab-bevacizumab arm in scenario analysis [33]. In terms of calibration of survival extrapolation, in NICE TA 474 and Saiyed et al., authors used external RWD of the matched GIDEON data and Australian Cancer Database to calibrate the OS extrapolations [47, 60].

Generally, in advanced HCC models, transition probabilities (n = 29, 53%) in Markov models and survival functions (n = 27, 49%) in partitioned survival models informed HCC progression (i.e. distribution of patients in each state in each cycle, Table 4). Concerning the transition probabilities, several approaches were adopted for those calculations in the included Markov models. Parametric survival modelling was the most frequently used method (n = 13, 24%), while model calibration (n = 2, 4%) [41, 56] and non-parametric count method (n = 1, 2%) [48] were also involved. In order to estimate the time-dependent transition probabilities in cancer models, the assumptions of parametric distributions for survival data were usually required. The Weibull distribution was often chosen since it belonged to the continuous probability distribution, of which the survival function parameters were then employed for deriving transition probabilities [70]. Five models using parametric survival modelling methods reported the following formulas of transition probabilities based on Weibull distribution:

$$TP\;\left(t\right)=1–\text{exp}\left[\lambda {\left(t-u\right)}^{\gamma }-\lambda t^{\gamma }\right],$$

λ = scale parameter of Weibull distribution, γ = shape parameter of Weibull distribution, u = cycle length [30]; on this basis, a simplified formula was derived:

$$TP=1-e^{-R},R=-\text{ln}\left[0.5\right]/\left(time\;to\;event/number\;of\;treatment\;cycles\right)$$

[17, 34, 57, 59].

**Table 4:** The methods of HCC progression across included decision models.

Study	Methods of HCC progression
Cabibbo et al. (2020) [28]	TPs
Cabibbo et al. (2022) [29]	TPs
Cai et al. (2020) [30]	TPs Approach used to estimate TPs: survival modelling *TP* (*t*) = 1 –exp[*λ*(*t* − *u*)^*γ*^ − *λt*^*γ*^] λ = scale parameter, γ = shape parameter, u = cycle length
Camma et al. (2013) [31]	TPs Approach used to estimate TPs: survival modelling
Carr et al. (2010) [15]	TPs
Chiang et al. (2020) [32]	TPs
Chiang et al. (2021) [33]	TPs
Elsisi et al. (2019) [19]	TPs Sorafenib: PFS-PD: 0.157 (0.125–0.188) PFS-death: 0.002 (0.002–0.003) PD-death: 0.500 (0.400–0.600) BSC: PFS-PD: 0.290 (0.232–0.349) PFS-death: 0.002 (0.002–0.003) PD-death: 0.219 (0.175–0.263) Distribution: gamma
Guan et al. (2022) [20]	Survival functions
Gupta et al. (2019) [18]	TPs Sorafenib: PFS-PD: 0.179 (95% CI: 0.158–0.199) PFS-death: 0.004 (95% CI: 0.003–0.004) PD-death: 0.375 (95% CI: 0.333–0.417) BSC: PFS-PD: 0.357 (95% CI: 0.317–0.398) PFS-death: 0.004 (95% CI: 0.003–0.004) PD-death: 0.412 (95% CI: 0.262–0.328) Distribution: beta
Ho et al. (2018) [34]	TPs Approach used to estimate TPs: survival modelling *TP* (1 *month*) = 1 –(0.5)^(1/*median time to event*)^ derived from: *TP* = 1 − *e*^−*R*^, *R* = −ln[0.5]/(*time to event*/*number of treatment cycles*) Sorafenib monotherapy: PFS-PD: 0.2264 PFS-death: 0.1097 PD-death: 0.1912 Sorafenib combination therapy: PFS-PD: 0.1399 PFS-death: 0.0739 PD-death: 0.1447 Distribution: beta
Hou et al. (2020) [35]	Survival functions
Ikeda et al. (2021) [36]	Survival functions
Kim et al. (2020) [37]	TPs Approach used to estimate TPs: survival modelling
Kobayashi et al. (2019) [38]	Survival functions
Li et al. (2021) [39]	TPs Approach used to estimate TPs: survival modelling
Li et al. (2022) [24]	Survival functions
Li et al. (2022) [40]	Survival functions
Liao et al. (2019) [41]	TPs Approach used to estimate TPs: model calibration Cabozantinib: PFS-PD: 0.091 (0.0637‐0.1183) PFS-death: 0.054 (0.0378‐0.0702) PD-death: 0.083 (0.0581‐0.1079) BSC: PFS-PD: 0.218 (0.1526‐0.2834) PFS-death: 0.082 (0.0574‐0.1066) PD-death: 0.093 (0.0651‐0.1209)
Liu et al. (2022) [42]	TPs and survival functions Approach used to estimate TPs: survival modelling
Meng et al. (2021) [43]	TPs and survival functions Approach used to estimate TPs: survival modelling
Meng et al. (2022) [23]	Survival functions
Meng et al. (2022) [21]	Survival functions
Meyers et al. (2021) [44]	Survival functions
Muszbek et al. (2008) [16]	TPs
NICE TA 474 (2017) [60]	TPs Approach used to estimate TPs: survival modelling
NICE TA 551 (2018) [61]	Survival functions
NICE TA 555 (2019) [62]	Survival functions
NICE TA 666 (2020) [63]	Survival functions
NICE TA 849 (2022) [64]	Survival functions
Parikh et al. (2017) [45]	TPs Assumed constant HCC progression rates over time
pCODR 10119 (2018) [65]	Survival functions
pCODR 10134 (2018) [66]	Survival functions
pCODR 10175 (2019) [67]	Survival functions
pCODR 10186 (2020) [68]	Survival functions
pCODR 10217 (2020) [69]	Survival functions
Peng et al. (2022) [25]	TPs
Qin et al. (2018) [46]	TPs
Saiyed et al. (2020) [47]	Survival functions
Sangmala et al. (2018) [48]	TPs Approach used to estimate TPs: non-parametric count method Sorafenib: 1^st^ line no progression-1^st^ line no progression: 0.4227 ± 0.0309 1^st^ line no progression-1^st^ line continued post progression: 0.2088 ± 0.0203 1^st^ line no progression-palliative care: 0.1955 ± 0.0198 1^st^ line no progression-death: 0.1731 1^st^ line continued post progression-1^st^ line continued post progression: 0.4451 1^st^ line continued post progression-palliative care: 0.4152 ± 0.0616 1^st^ line continued post progression-death: 0.5848 Palliative care-palliative care: 0.4152 ± 0.0616 Palliative care-death: 0.1397 ± 0.0096 Palliative care: 1^st^ line no progression-1^st^ line no progression: 0.7330 1^st^ line no progression-palliative care post-progression: 0.1520 ± 0.0026 1^st^ line no progression-death: 0.1150 ± 0.0045 Palliative care post-progression-palliative care post-progression: 0.7808 Palliative care post-progression-death: 0.2192 ± 0.0030
Sherrow et al. (2020) [49]	TPs
Shi et al. (2021) [50]	Survival functions
Shlomai et al. (2018) [51]	TPs Approach used to estimate TPs: survival modelling
Shlomai et al. (2019) [52]	TPs Approach used to estimate TPs: survival modelling
Sieg et al. (2020) [53]	TPs $\text{PFS-PFS}:e^{\;\frac{yPFS}{number\;of\;treatment\;cycles}}$ $\text{PFS-death}:1\;-\;e^{\;\frac{yOS}{number\;of\;treatment\;cycles}}$ $\text{PD-death}:1\;-\;e^{\;\frac{yOS-yPFS}{number\;of\;treatment\;cycles}}$
Soto-Perez-de-Celis et al. (2019) [54]	-
Su et al. (2021) [55]	Survival functions
Wen et al. (2021) [56]	TPs Approach used to estimate TPs: model calibration Atezolizumab-bevacizumab: PFS-PD: 0.0656 PFS-death: 0.0263 PD-death: 0.0495 Sorafenib: PFS-PD: 0.1159 PFS-death: 0.0398 PD-death: 0.0813
Zhang et al. (2015) [17]	TPs Approach used to estimate TPs: survival modelling *TP* (1 *month*) = 1 –(0.5)^(1/*median time to event*)^ derived from: *TP* = 1 − *e*^−*R*^, *R* = −ln[0.5]/(*time to event*/*number of treatment cycles*) Sorafenib: PFS-PD: 0.143 PFS-death: 0.083 PD-death: 0.180 BSC: PFS-PD: 0.390 PFS-death: 0.152 PD-death: 0.219
Zhang et al. (2016) [57]	TPs Approach used to estimate TPs: survival modelling *TP* (1 *month*) = 1 –(0.5)^(1/*median time to event*)^ derived from: *TP* = 1 − *e*^−*R*^, *R* = −ln[0.5]/(*time to event*/*number of treatment cycles*) FOLFOX4: PFS-PFS: 0.686 PFS-PD: 0.211 PFS-death: 0.103 PD-PD: 0.819 PD-death: 0.181 Sorafenib: PFS-PFS: 0.680 PFS-PD: 0.219 PFS-death: 0.101 PD-PD: 0.829 PD-death: 0.171
Zhang et al. (2021) [58]	Survival functions
Zhao et al. (2022) [22]	Survival functions
Zheng et al. (2020) [59]	TPs Approach used to estimate TPs: survival modelling *TP* (1 *month*) = 1 –(0.5)^(1/*median time to event*)^ derived from: *TP* = 1 − *e*^−*R*^, *R* = −ln[0.5]/(*time to event*/*number of treatment cycles*) Ramucirumab: PFS-PFS: 0.703 PFS-PD: 0.219 PFS-death: 0.078 PD-PD: 0.885 PD-death: 0.115 Placebo: PFS-PFS: 0.557 PFS-PD: 0.352 PFS-death: 0.091 PD-PD: 0.885 PD-death: 0.115
Zhou et al. (2022) [26]	Survival functions
Zhou et al. (2022) [27]	Survival functions

Abbreviations: BSC, best supportive care; HCC, hepatocellular carcinoma; NICE, National Institute for Health and Care Excellence; OS, overall survival; pCODR, pan-Canadian Oncology Drug Review; PD, progressive disease; PFS, progression-free survival; TA, technology appraisal; TPs, transition probabilities.

The remaining 24 Markov models did not clarify how the transition probabilities were obtained. Furthermore, for reducing calculation, the probability from PFS state to death was commonly assumed to be the natural mortality in most models, and the constant transition probabilities over time were even assumed in 10 ones [17–19, 34, 41, 45, 48, 56, 57, 59].

#### Utility and cost inputs

Table G in S1 Appendix presented the utility and cost inputs of identified models. Two approaches were reported for utility modelling (Table 5). A total of 51 studies (93%) adopted the state-based approach, whereas only one study (2%) modelled utilities through the proximity to death approach in base-case analysis [63]. The studies using a state-based approach defined the utilities of PFS and PD states. One study pursued the following time-to-death intervals when applying this approach: > 30 weeks, > 15 to ≤ 30 weeks, > 5 to ≤ 15 weeks, and ≤ 5 weeks before death [63].

**Table 5:** Key characteristics of utility and cost inputs.

Study	Approach to utility modelling	Consideration of AEs in utility and cost inputs
Cabibbo et al. (2020) [28]	-	-
Cabibbo et al. (2022) [29]	-	-
Cai et al. (2020) [30]	State based	Yes (disutility and cost)
Camma et al. (2013) [31]	State based	No
Carr et al. (2010) [15]	-	Yes (cost)
Chiang et al. (2020) [32]	State based	Yes (disutility and cost)
Chiang et al. (2021) [33]	State based	Yes (disutility and cost)
Elsisi et al. (2019) [19]	State based	Yes (cost)
Guan et al. (2022) [20]	State based	Yes (disutility and cost)
Gupta et al. (2019) [18]	State based	Yes (cost)
Ho et al. (2018) [34]	State based	No
Hou et al. (2020) [35]	State based	Yes (disutility and cost)
Ikeda et al. (2021) [36]	State based	Yes (cost)
Kim et al. (2020) [37]	State based	Yes (disutility and cost)
Kobayashi et al. (2019) [38]	State based	Yes (disutility and cost)
Li et al. (2021) [39]	State based	Yes (disutility and cost)
Li et al. (2022) [24]	State based	Yes (disutility and cost)
Li et al. (2022) [40]	State based	Yes (disutility and cost)
Liao et al. (2019) [41]	State based	Yes (cost)
Liu et al. (2022) [42]	State based	Yes (disutility and cost)
Meng et al. (2021) [43]	State based	Yes (cost)
Meng et al. (2022) [23]	State based	Yes (disutility and cost)
Meng et al. (2022) [21]	State based	Yes (disutility and cost)
Meyers et al. (2021) [44]	State based	Yes (cost)
Muszbek et al. (2008) [16]	-	Yes (cost)
NICE TA 474 (2017) [60]	State based	Yes (disutility and cost)
NICE TA 551 (2018) [61]	State based	No/NR
NICE TA 555 (2019) [62]	State based	Yes (disutility and cost)
NICE TA 666 (2020) [63]	TTD or State based	Yes (disutility and cost)
NICE TA 849 (2022) [64]	State based	Yes (disutility and cost)
Parikh et al. (2017) [45]	State based	Yes (disutility and cost)
pCODR 10119 (2018) [65]	State based	Yes (disutility and cost)
pCODR 10134 (2018) [66]	State based	Yes (disutility and cost)
pCODR 10175 (2019) [67]	State based	Yes (disutility and cost)
pCODR 10186 (2020) [68]	State based	No/NR
pCODR 10217 (2020) [69]	State based	Yes (disutility and No/NR)
Peng et al. (2022) [25]	State based	Yes (disutility and cost)
Qin et al. (2018) [46]	State based	Yes (cost)
Saiyed et al. (2020) [47]	State based	Yes (disutility and cost)
Sangmala et al. (2018) [48]	State based	Yes (cost)
Sherrow et al. (2020) [49]	State based	No/NR
Shi et al. (2021) [50]	State based	Yes (cost)
Shlomai et al. (2018) [51]	State based	Yes (disutility and cost)
Shlomai et al. (2019) [52]	State based	Yes (disutility and cost)
Sieg et al. (2020) [53]	State based	Yes (disutility and cost)
Soto-Perez-de-Celis et al. (2019) [54]	State based	Yes (disutility and cost)
Su et al. (2021) [55]	State based	Yes (disutility and cost)
Wen et al. (2021) [56]	State based	Yes (cost)
Zhang et al. (2015) [17]	State based	Yes (cost)
Zhang et al. (2016) [57]	State based	Yes (cost)
Zhang et al. (2021) [58]	State based	Yes (cost)
Zhao et al. (2022) [22]	State based	Yes (disutility and cost)
Zheng et al. (2020) [59]	State based	Yes (cost)
Zhou et al. (2022) [26]	State based	Yes (disutility and cost)
Zhou et al. (2022) [27]	State based	Yes (disutility and cost)

Abbreviations: AE, adverse event; NICE, National Institute for Health and Care Excellence; NR, not reported; pCODR, pan-Canadian Oncology Drug Review; TA, technology appraisal; TTD, time to death.

The utilities of 0.76 and 0.68 used for PFS and PD states were the most common values for advanced HCC models (n = 27, 49%), and the pCODR Review Team considered these utility values more appropriate for the advanced HCC patient population [66]. In 17 studies (31%), utility values were equivalent across the treatment arms, since there was a lack of exploration for differentiation possibility or consideration for disutility of adverse events in base-case or scenario analysis. Thirty-three studies (60%) assumed different utilities across the treatment arms by means of applying the disutilities of adverse events or differentiating the values of base utility. Additionally, one study (2%) also used age adjustment to utility values [64].

With regard to the sources of utility, 33 studies (60%) generated the utility values only from published literature. Fifteen studies (27%) applied the EQ-5D utilities obtained in the trials, of which one (2%) used the adjusted EQ-5D data from REFLECT to reflect Japan-specific utilities [36]. Two studies (4%) involved the FACT-Hep data collected during clinical trials [45, 60] and one (2%) reported the use of SF-6D data from Chinese HCC patients [48].

The majority of studies applied the healthcare system perspective in base-case analyses (n = 28, 51%), followed by the payer (n = 22, 40%), societal (n = 4, 7%) [18, 40, 57, 67], hospital [19], and patient [57] (each n = 1, 2%) perspectives. Most studies only considered direct medical costs (n = 46, 84%), whereas two studies (4%) also included indirect costs, such as salary or patient time loss, caregiver, and transportation costs [40, 57]. Costs of management of adverse events were considered in 47 studies (85%, Table 5).

#### Sensitivity analyses and validation efforts

The most common sensitivity analyses for advanced HCC models were one-way sensitivity analysis (n = 47, 85%), probabilistic sensitivity analysis (n = 49, 89%), and scenario analysis (n = 30, 55%). Other sensitivity analysis methods, including two-way sensitivity analysis and subgroup analysis, were reported by six studies (11%) and nine studies (16%), respectively (Table 3).

Five types of model validation were observed in 13 studies (24%). Face validity was the most frequently described validation effort (n = 12, 22%), followed by internal validity (n = 4, 7%) [31, 60, 63, 64], cross-validation (n = 3, 5%) [47, 63, 64], external validation (n = 3, 5%) [47, 60, 63], and calibration (n = 2, 4%, Table 3) [41, 56].

## Discussion

This systematic literature review was the first study to evaluate disease modelling for health economic evaluations of systemic therapies in advanced HCC, focusing on methodological characteristics and key challenges of decision models. This systematic analysis comprised modelling technique and structure, time horizon, cycle length, discounting, clinical effectiveness inputs, utility and cost inputs, sensitivity analyses, and validation efforts. It revealed wide variations of economic evaluation results in advanced HCC modelling studies, which could be attributed to marked differences in the adopted model approaches and assumptions. Consequently, the considerations and selections of (1) model structure and technique, (2) time horizon, cycle length and discounting, (3) clinical effectiveness inputs, (4) utility and cost inputs, (5) sensitivity analyses and validation efforts, (6) patient characteristics, (7) duration of treatment, and (8) switch, were discussed in this section. The key recommendations proposed across the discussion are summarized in Table 6.

**Table 6:** Summary of key recommendations for disease modelling for health economic evaluations of systemic therapies in advanced HCC.

Category	Summary of recommendations
***General recommendations***	
Reporting good practices	To improve reporting good practices, especially for selection of extrapolation methods, decisions on measurement of effectiveness, identification of parameters used in sensitivity analysis and assumption of distributions or uncertainty for these parameters
Modelling good practices	To improve modelling good practices, especially for definition of model structure that reflects the clinical practice, selection of modelling technique that matches the structure, explanation of time-to-event parameters (including distribution parameters and transition probabilities), selection of approach to utility modelling, presentation of methods of sensitivity analyses, utilization of model validation
***Specific recommendations***	
Model structure	To distinguish different treatment lines in the model structure to simulate more realistic treatment pathways
Modelling technique	To adopt the patient-level modelling technique if available which can provide flexibility in the fields of event timing, competing event handling, and model structure
Time horizon	To use a lifetime horizon
Extrapolation	To appropriately adopt external data and trial data-based parametric distribution for OS extrapolation to balance the external and internal validity
Utility inputs	To consider the quality of available data when selecting utility modelling approach, and to consider the disutilities of adverse events
Heterogeneity	To analyze relevant identifiable subgroups according to different patient characteristics
Patient characteristics	To evaluate the differences of patient characteristics, and to consider the modelling being more sensitive to these differences
Duration of treatment	To model duration of treatment using time to discontinuation, when the Kaplan–Meier curve or IPD of time to discontinuation are available
Treatment switching	To employ and define the most suitable adjustment method matching the corresponding assumptions, and to compare adjusted and actual OS curves visually

Abbreviations: HCC, hepatocellular carcinoma; IPD, individual patient data; OS, overall survival.

### Model structure and technique

Most advanced HCC models used the traditional three health states of PFS, PD, and death to define their structures. However, applying such a structure would be problematic if the models were to consider conditional outcomes and treatment sequences. Most health-state-driven models accounted for all subsequent therapy in a PD state, implying patients received the same treatment after disease progression until death. This assumption did not reflect the clinical practice, and stronger ones were required for modelling downstream treatments. Thus, combining treatment-line-driven structure could be more realistic in simulating treatment pathways.

In the economic evaluations for oncology therapies, the dominant decision analysis models involved Markov and partitioned survival models, which were the only techniques for modelling the health and economic outcomes in included publications and TAs. However, when considering multiple adverse events and different treatment lines, a number of assumptions and model states were required, which would contribute to greatly untransparent and complex models. Therefore, it is more suggested to adopt patient-level modelling techniques including patient-level long short-term memory or discrete event simulation. Specially, discrete event simulation could provide flexibility in the fields of event timing, competing event handling, and model structure, leading to a preferable reflection of clinical practice. Patient-level techniques, however, were widely regarded as more time consuming and complex, and there was lack of guidance for model implementation.

### Time horizon, cycle length, and discounting

Time horizons, cycle lengths, and discount rates differed between studies in this review. NICE guideline for TA recommends that the health economic model should adopt a sufficiently long time horizon to evaluate all significant differences in benefits and survivals between compared treatments [71]. When alternative treatments impact outcomes or costs that persist throughout a patient’s remaining life, a horizon of lifetime is usually required [71]. In addition, the cycle length is generally accepted to be consistent with the treatment cycle. The annual discount rate changes over time and across jurisdictions, such as currently 3.5% recommended for UK [71], 5% for China [72], and 5% for Australia [73].

### Clinical effectiveness inputs

The research community seemed to hardly get access to IPD from industry-funded trials, which led to limitations in decision making, structure employed, and methods used. OS served as the critical parameter impacting on economic evaluation results, but its extrapolation was filled with challenges for the treatments associated with sustained responses. It was difficult to accurately model the OS only adopting parametric distributions based on trial data, since a plateau of the tail occurred in the sustained response-related OS curve. In accordance with guidelines of HTA, the extrapolation and plausibility of long-term survival can be informed and assessed using external data [74]. The external and internal validity are standardly balanced through trade-off between adopting external data and trial data-based parametric distribution. Importantly, the treatment options and patient population of external data should be assessed to coincide with that in the trial. We found only one study using SEER data for the extrapolation of OS in a pessimistic scenario [33]. Moreover, calibrating the extrapolation based on external data becomes a good practice. We identified two studies using RWD (i.e. the matched GIDEON data and Australian Cancer Database) for calibration of their parametric OS extrapolations [47, 60].

When adopting state transition models, there was a rising need for justifications of transition probabilities that were used between states. The most frequently used approach for cancer models in estimating transition probabilities was parametric survival modelling methods [75]. The maximum likelihood estimation was typically used during parametric survival functions fitting to IPD for estimations of survival function parameters, such as the shape and scale parameters of the Weibull distribution, which were employed to derive transition probabilities later. The transition probabilities of the interest events were defined as:

$$TP=1–S\left(t_{i}\right)/S\left(t_{i-1}\right),$$

S = survivor function, (t_i-1_, t_i_) = time interval [75].

In fact, most Markov models of systemic therapies in advanced HCC did not describe the calculation method or formula derivation process for transition probabilities, which could be associated with the major uncertainty and become one of the concerns in future models.

### Utility and cost inputs

Utility inputs significantly affect economic evaluation results. Given the utility models, the data quality has an important impact on the approach adopted. For instance, there should be enough patient samples in each proximity-to-death interval and the utility value should be validated for its face validity, if the time-to-death approach is taken. In NICE TA 666, the decision model defined the utilities based on time-to-death intervals, because this approach could drive a more refined division of health states compared with the state-based approach [63]. However, the evidence review groups from NICE did not clearly favor time-to-death or state-based utility models, and thus further evidence should be needed.

It was also worth noting that adverse events were generally served as an important part of the cost and utility in a treatment cycle, but seldom involved as separate events. Consequently, the costs of managing adverse events and disutilities of adverse events deserve attention and research in advanced HCC models.

### Sensitivity analyses and validation efforts

The studies provided limited details on the methodologies of sensitivity analyses, even though more than 90% of them reported one-way or probabilistic sensitivity analyses. Particularly in probabilistic analysis, identifying included parameters and quantifying uncertainty surrounding them were extremely important; otherwise, interpreting adopted confidence intervals was unlikely.

For only 13 studies, five types of validation were used in the publication, mainly being face validity of models and assumptions judged by experts. The validity and outcomes of models could be questioned if being a lack of model validation. Thus, future health economic models for advanced HCC need to better utilize the existing frameworks to reflect the validity of the models [76, 77].

### Patient characteristics

The differences in epidemiological and physiological characteristics, including age, sex, ethnicity, Eastern Cooperative Oncology Group performance status, etiology of disease, Child-Pugh class, Barcelona Clinic Liver Cancer (BCLC) stage, extrahepatic spread of disease, macrovascular invasion, alpha-fetoprotein concentration, and previous treatment history in patients diagnosed as advanced HCC, could result in needs for modelling which is more associated with these differences. Actually, the identified models rarely took this into consideration, and thus future studies were required to consider the modelling being more sensitive to the differences of patient characteristics, and to categorize advanced HCC patients into different appropriate models based on different patient characteristics and evaluate them separately.

### Duration of treatment

There was an ongoing challenge to determine the optimal duration of systemic therapies for advanced HCC in clinical practice. Duration of treatment was modelled mostly adopting time to discontinuation and PFS. The data of time to discontinuation would be preferable against that of PFS, when the Kaplan–Meier curve or IPD of time to discontinuation were available. Otherwise, PFS could be considered as a proxy of time to discontinuation. However, given that patients might continue on previous treatment after disease progression, this approach might underestimate the treatment duration likely presented in clinical practice. Most advanced HCC models did not define the measure of treatment duration, and this concern should be solved in the future.

### Switch

Treatment switching due to disease progression or intolerance commonly occurred in the trials for advanced HCC and its pattern, namely proportion of switching and treatments, needed to be reflective of clinical practice in the analyzed jurisdiction. The majority of identified studies involved treatment switching, yet few presented the selected adjustment methods and rationales, implying poor quality for reporting the switching adjustment implementation [78]. A technical support document from NICE [79] and a publication [78] provided definitions and applications of various switching adjustment methods such as inverse probability of censoring weights and iterative parameter estimation algorithm. Future studies should employ the most suitable adjustment method matching the corresponding assumptions and visually comparing adjusted and actual OS curves [78, 79].

### Limitations

There were certain limitations in this review. Firstly, owing to the absence of a more appropriate alternative, we used the CHEERS 2022 checklist that was not designed to assess publication quality. The CHEERS checklist scores were not generally reflective of the modelling quality, since reporting on items did not actually represent that they were appropriately implemented. Secondly, some extracted data lacked definite data sources, model structures, assumptions, and adopted methods, and readers and researchers might not agree with these categorizations. To reduce such impact to the utmost extent, two other authors verified all the extracted information.

## Conclusions

Disease modelling for health economic evaluations of systemic therapies in advanced HCC has adopted various modelling approaches and assumptions, resulting in marked differences in economic evaluation results. It implied that there had been much uncertainty associated with cost-effectiveness of systemic therapies for HCC. By proposing methodological recommendations, we suggest that future model-based studies for health economic evaluation of HCC therapies should follow good modelling practice guidelines and improve modelling methods to generate reliable health and economic evidence, especially in definition of model structure that reflects the clinical practice, selection of modelling technique that matches the structure, explanation of time-to-event parameters, selection of approach to utility modelling, presentation of methods of sensitivity analyses, and utilization of model validation.

## Supporting information

S1 Appendix Supporting tables. (DOCX)

S1 Checklist PRISMA 2020 checklist. (DOCX)

## Abbreviations

BCLC: Barcelona Clinic Liver Cancer
BSC: best supportive care
CADTH: Canadian Agency for Drugs and Technologies in Health
CHEERS: Consolidated Health Economic Evaluation Reporting Standards
CNKI: China National Knowledge Infrastructure
HCC: hepatocellular carcinoma
HTA: health technology assessment
ICI: immune checkpoint inhibitor
IPD: individual patient data
LYG: life-year gained
NICE: National Institute for Health and Care Excellence
OS: overall survival
pCODR: pan-Canadian Oncology Drug Review
PD: progressive disease
PFS: progression-free survival
PRISMA: Preferred Reporting Items for Systematic Reviews and Meta-Analyses
QALY: quality-adjusted life-year
RCT: randomized controlled trial
RFS: recurrence-free survival
RWD: real-world data
SEER: Surveillance, Epidemiology, and End Results
TA: technology appraisal
TKI: tyrosine kinase inhibitor
US: United States
VEGF: vascular endothelial growth factor
