# Supplementary material for: Comparative analysis of disease modelling for health economic evaluations of systemic therapies in advanced hepatocellular carcinoma
Source: PLoS One. 2023 Oct 5;18(10):e0292239. doi: 10.1371/journal.pone.0292239 (PMC10553296; doi:10.1371/journal.pone.0292239)
Supplement: S1 Appendix — (DOCX) [file pone.0292239.s001.docx]

**S1 Appendix**

***Comparative analysis of disease modelling for health economic evaluations of systemic therapies in advanced hepatocellular carcinoma***

Huimin Zou^1^, Yan Xue^1^, Xianwen Chen^1^, Yunfeng Lai^2^, Dongning Yao^3^, Carolina Oi Lam Ung^1,4,5^, Hao Hu^1,4,5*^

^1^ State Key Laboratory of Quality Research in Chinese Medicine, Institute of Chinese Medical Sciences, University of Macau, Macao SAR, China

^2^ School of Public Health and Management, Guangzhou University of Chinese Medicine, Guangzhou, China

^3^ Department of Drug Regulatory Science and Pharmacoeconomics, School of Pharmacy, Nanjing Medical University, Nanjing, China

^4^ Centre for Pharmaceutical Regulatory Sciences, University of Macau, Macao SAR, China

^5^ Department of Public Health and Medicinal Administration, Faculty of Health Sciences, University of Macau, Macao SAR, China

* Corresponding author

E-mail: haohu@um.edu.mo (HH)

**S1A Table. Eligibility criteria.**

| **Inclusion criteria** | |
| --- | --- |
| Population | Adult patients (aged ≥ 18 years) with advanced, metastatic or unresectable HCC |
| Intervention | Any types of systemic therapies for population of interest |
| Comparator | Any types of systemic therapies, BSC and/or placebo |
| Setting, country | All countries, all settings |
| Outcomes | EEs: cost-utility results, cost-effectiveness results, cost-benefit results, cost-minimization results, cost-consequence results, risk-benefit results |
| Study design | Model-based full EEs, such as cost-utility, cost-effectiveness, cost-benefit, cost-minimization, cost-consequence, or risk-benefit analyses |
| **Exclusion criteria** | |
| Types of EEs | Partial EEs, trial-based evaluations |
| Language restrictions | Not English or Chinese |
| Publication type | Conference abstracts, commentaries, editorials, reviews |
| Study design | Animal studies, *in vitro* studies |

Abbreviations: BSC, best supportive care; EE, economic evaluation; HCC, hepatocellular carcinoma.

**S1B Table. Search strategies.**

| **Item** | **Search** |
| --- | --- |
| 1 | ((liver or hepat*) adj3 (neoplas* or cancer* or tumor* or tumour* or carcinoma* or oncolog* or malign*)).mp. |
| 2 | (economic adj3 (model* or analy* or assess* or evaluat*)).mp. |
| 3 | (pharmacoeconomic* adj3 (model* or analy* or assess* or evaluat*)).mp. |
| 4 | (cost adj3 (util* or effective* or efficac* or benef* or minim* or conseq*)).mp. |
| 5 | "cost utility analysis"/ |
| 6 | "cost effectiveness analysis"/ |
| 7 | "cost benefit analysis"/ |
| 8 | 2 or 3 or 4 or 5 or 6 or 7 |
| 9 | 1 and 8 |

**S1C Table. Assessment of the CHEERS 2022 checklist on study level.**

| **Publication** | **Title** | **Abstract** | **Background and objectives** | **Health economic analysis plan** | **Study population** | **Setting and location** | **Comparators** | **Perspective** | **Time horizon** | **Discount rate** | **Selection of outcomes** | **Measurement of comes** | **Valuation of outcomes** | **Measurement and valuation of resources and costs** | **Currency, price date, and conversion** | **Rationale and description of model** | **Analytics and assumptions** | **Characterizing heterogeneity** | **Characterizing distributional effects** | **Characterizing uncertainty** | **Approach to engagement with patients and others affected by the study** | **Study parameters** | **Summary of main results** | **Effect of uncertainty** | **Effect of engagement with patients and others affected by the study** | **Study findings, limitations, generalizability, and current knowledge** | **Source of funding** | **Conflicts of interest** | **Score** |
| --- | --- | --- | --- | --- | --- | --- | --- | --- | --- | --- | --- | --- | --- | --- | --- | --- | --- | --- | --- | --- | --- | --- | --- | --- | --- | --- | --- | --- | --- |
| Cabibbo et al. (2020) | 0 | 1 | 1 | 0 | 1 | 0 | 1 | - | 1 | 0 | 1 | 1 | 1 | - | - | 1 | 1 | 0 | 0 | 1 | 0 | 0 | 1 | 1 | 0 | 1 | 1 | 1 | 64% |
| Cabibbo et al. (2021) | 0 | 1 | 1 | 0 | 1 | 0 | 1 | - | 1 | 0 | 1 | 1 | 1 | - | - | 1 | 1 | 0 | 0 | 1 | 0 | 1 | 1 | 1 | 0 | 1 | 1 | 1 | 68% |
| Cai et al. (2020) | 1 | 1 | 1 | 0 | 1 | 1 | 1 | 1 | 1 | 1 | 1 | 0 | 1 | 1 | 1 | 1 | 1 | 0 | 0 | 1 | 0 | 0 | 1 | 1 | 0 | 1 | 1 | 1 | 75% |
| Camma et al. (2013) | 0 | 0 | 1 | 0 | 1 | 1 | 1 | 1 | 1 | 1 | 1 | 1 | 1 | 1 | 1 | 1 | 1 | 0 | 0 | 1 | 0 | 0 | 1 | 1 | 0 | 1 | 0 | 1 | 68% |
| Carr et al. (2010) | 0 | 1 | 1 | 0 | 1 | 1 | 1 | 1 | 1 | 1 | 1 | 1 | 1 | 1 | 1 | 1 | 1 | 0 | 0 | 1 | 0 | 1 | 1 | 1 | 0 | 1 | 1 | 1 | 79% |
| Chiang et al. (2021) | 0 | 1 | 1 | 0 | 1 | 1 | 1 | 1 | 1 | 1 | 1 | 0 | 1 | 1 | 1 | 1 | 1 | 1 | 0 | 1 | 0 | 1 | 1 | 1 | 0 | 1 | 0 | 1 | 75% |
| Chiang et al. (2021) | 1 | 1 | 1 | 0 | 1 | 1 | 1 | 1 | 1 | 1 | 1 | 1 | 1 | 1 | 1 | 1 | 1 | 1 | 0 | 1 | 0 | 1 | 1 | 1 | 0 | 1 | 1 | 1 | 86% |
| Elsisi et al. (2018) | 1 | 1 | 1 | 0 | 1 | 1 | 1 | 1 | 1 | 1 | 1 | 1 | 1 | 1 | 1 | 1 | 1 | 0 | 0 | 1 | 0 | 1 | 1 | 1 | 0 | 1 | 1 | 1 | 82% |
| Guan et al. (2022) | 0 | 1 | 1 | 0 | 1 | 1 | 1 | 1 | 1 | 1 | 1 | 0 | 1 | 1 | 1 | 1 | 1 | 0 | 0 | 1 | 0 | 1 | 1 | 1 | 0 | 1 | 1 | 1 | 75% |
| Gupta et al. (2018) | 0 | 1 | 1 | 0 | 1 | 1 | 1 | 1 | 1 | 1 | 1 | 0 | 1 | 1 | 1 | 1 | 0 | 0 | 0 | 1 | 0 | 1 | 1 | 1 | 0 | 1 | 0 | 1 | 68% |
| Ho et al. (2018) | 1 | 1 | 1 | 0 | 1 | 1 | 1 | 1 | 1 | 1 | 1 | 0 | 1 | 1 | 1 | 1 | 1 | 0 | 0 | 1 | 0 | 1 | 1 | 1 | 0 | 1 | 1 | 1 | 79% |
| Hou et al. (2020) | 1 | 0 | 1 | 0 | 1 | 1 | 1 | 1 | 0 | 1 | 1 | 0 | 1 | 1 | 1 | 1 | 1 | 1 | 0 | 1 | 0 | 1 | 1 | 1 | 0 | 1 | 1 | 1 | 75% |
| Ikeda et al. (2021) | 0 | 1 | 1 | 0 | 1 | 1 | 1 | 1 | 1 | 1 | 1 | 0 | 1 | 1 | 1 | 1 | 1 | 0 | 0 | 1 | 0 | 0 | 1 | 1 | 0 | 1 | 1 | 1 | 71% |
| Kim et al. (2019) | 1 | 1 | 1 | 0 | 1 | 1 | 1 | 1 | 1 | 1 | 1 | 1 | 1 | 1 | 1 | 1 | 1 | 1 | 0 | 1 | 0 | 0 | 1 | 1 | 0 | 1 | 1 | 1 | 82% |
| Kobayashi et al. (2019) | 1 | 1 | 1 | 0 | 1 | 1 | 1 | 1 | 1 | 1 | 1 | 0 | 1 | 1 | 1 | 1 | 1 | 0 | 0 | 1 | 0 | 1 | 1 | 1 | 0 | 1 | 0 | 1 | 75% |
| Li et al. (2021) | 0 | 1 | 1 | 0 | 1 | 1 | 1 | 1 | 1 | 1 | 1 | 0 | 1 | 1 | 1 | 1 | 1 | 0 | 0 | 1 | 0 | 1 | 1 | 1 | 0 | 1 | 1 | 1 | 75% |
| Li et al. (2022) | 0 | 1 | 1 | 0 | 1 | 1 | 1 | 1 | 1 | 1 | 1 | 0 | 1 | 1 | 1 | 1 | 1 | 0 | 0 | 1 | 0 | 1 | 1 | 1 | 0 | 1 | 0 | 1 | 71% |
| Li et al. (2022) | 1 | 1 | 1 | 0 | 1 | 1 | 1 | 1 | 1 | 1 | 1 | 0 | 1 | 1 | 1 | 1 | 1 | 1 | 0 | 1 | 0 | 1 | 1 | 1 | 0 | 1 | 1 | 1 | 82% |
| Liao et al. (2019) | 0 | 1 | 1 | 0 | 1 | 1 | 1 | 1 | 1 | 1 | 1 | 1 | 1 | 1 | 1 | 1 | 0 | 0 | 0 | 1 | 0 | 1 | 1 | 1 | 0 | 1 | 1 | 1 | 75% |
| Liu et al. (2022) | 1 | 1 | 1 | 0 | 1 | 1 | 1 | 1 | 1 | 1 | 1 | 0 | 1 | 1 | 0 | 1 | 1 | 0 | 0 | 1 | 0 | 1 | 1 | 1 | 0 | 1 | 1 | 0 | 71% |
| Meng et al. (2021) | 0 | 1 | 1 | 0 | 1 | 1 | 1 | 1 | 1 | 1 | 1 | 0 | 1 | 1 | 0 | 1 | 1 | 0 | 0 | 1 | 0 | 1 | 1 | 1 | 0 | 1 | 1 | 0 | 68% |
| Meng et al. (2022) | 1 | 1 | 1 | 0 | 1 | 1 | 1 | 1 | 1 | 1 | 1 | 0 | 1 | 1 | 1 | 1 | 1 | 0 | 0 | 1 | 0 | 1 | 1 | 1 | 0 | 1 | 0 | 1 | 75% |
| Meng et al. (2022) | 1 | 1 | 1 | 0 | 1 | 1 | 1 | 1 | 1 | 1 | 1 | 0 | 1 | 1 | 1 | 1 | 1 | 0 | 0 | 1 | 0 | 1 | 1 | 1 | 0 | 1 | 1 | 1 | 79% |
| Meyers et al. (2021) | 0 | 0 | 1 | 0 | 1 | 1 | 1 | 1 | 1 | 1 | 1 | 0 | 1 | 1 | 1 | 1 | 1 | 0 | 0 | 1 | 0 | 0 | 1 | 1 | 0 | 1 | 1 | 1 | 68% |
| Muszbek et al. (2008) | 0 | 1 | 1 | 0 | 1 | 1 | 1 | 1 | 1 | 1 | 1 | 1 | 1 | 1 | 1 | 1 | 1 | 0 | 0 | 1 | 0 | 1 | 1 | 1 | 0 | 1 | 1 | 1 | 79% |
| Parikh et al. (2017) | 0 | 1 | 1 | 0 | 1 | 1 | 1 | 1 | 0 | 1 | 1 | 1 | 1 | 1 | 1 | 1 | 0 | 0 | 0 | 1 | 0 | 0 | 1 | 1 | 0 | 1 | 1 | 1 | 68% |
| Peng et al. (2022) | 1 | 1 | 1 | 0 | 1 | 1 | 1 | 1 | 1 | 1 | 1 | 0 | 1 | 1 | 1 | 1 | 1 | 0 | 0 | 1 | 0 | 1 | 1 | 1 | 0 | 1 | 1 | 1 | 79% |
| Qin et al. (2018) | 1 | 1 | 1 | 0 | 1 | 1 | 1 | 1 | 1 | 1 | 1 | 0 | 1 | 1 | 0 | 1 | 0 | 0 | 0 | 1 | 0 | 1 | 1 | 1 | 0 | 1 | 1 | 1 | 71% |
| Saiyed et al. (2020) | 1 | 1 | 1 | 0 | 1 | 1 | 1 | 1 | 1 | 1 | 1 | 0 | 1 | 1 | 1 | 1 | 1 | 0 | 0 | 1 | 0 | 1 | 1 | 1 | 0 | 1 | 1 | 1 | 79% |
| Sangmala et al. (2018) | 0 | 1 | 1 | 0 | 1 | 1 | 1 | 1 | 1 | 1 | 1 | 0 | 1 | 1 | 1 | 1 | 1 | 0 | 0 | 1 | 0 | 1 | 1 | 1 | 0 | 1 | 0 | 1 | 71% |
| Sherrow et al. (2020) | 1 | 1 | 1 | 0 | 1 | 1 | 1 | 0 | 0 | 0 | 1 | 1 | 1 | 1 | 0 | 1 | 0 | 0 | 0 | 1 | 0 | 0 | 1 | 1 | 0 | 1 | 1 | 1 | 61% |
| Shi et al. (2021) | 0 | 1 | 1 | 0 | 1 | 1 | 1 | 1 | 1 | 1 | 1 | 0 | 1 | 1 | 0 | 1 | 1 | 0 | 0 | 1 | 0 | 1 | 1 | 1 | 0 | 1 | 0 | 0 | 64% |
| Shlomai et al. (2018) | 0 | 1 | 1 | 0 | 1 | 1 | 1 | 1 | 0 | 1 | 1 | 1 | 1 | 1 | 1 | 1 | 1 | 0 | 0 | 1 | 0 | 1 | 1 | 1 | 0 | 1 | 1 | 1 | 75% |
| Shlomai et al. (2019) | 0 | 1 | 1 | 0 | 1 | 1 | 1 | 1 | 1 | 1 | 1 | 0 | 1 | 1 | 1 | 1 | 1 | 0 | 0 | 1 | 0 | 1 | 1 | 1 | 0 | 1 | 1 | 1 | 75% |
| Sieg et al. (2020) | 0 | 1 | 1 | 0 | 1 | 1 | 1 | 1 | 1 | 1 | 1 | 0 | 1 | 1 | 1 | 1 | 1 | 0 | 0 | 1 | 0 | 1 | 1 | 1 | 0 | 1 | 1 | 1 | 75% |
| Soto-Perez-de-Celis et al. (2019) | 0 | 1 | 1 | 0 | 1 | 1 | 1 | 1 | 0 | 0 | 1 | 0 | 1 | 1 | 1 | 0 | 0 | 0 | 0 | 1 | 0 | 1 | 1 | 1 | 0 | 1 | 1 | 0 | 57% |
| Su et al. (2021) | 1 | 1 | 1 | 0 | 1 | 1 | 1 | 1 | 0 | 0 | 1 | 0 | 1 | 1 | 1 | 1 | 1 | 1 | 0 | 1 | 0 | 1 | 1 | 1 | 0 | 1 | 1 | 1 | 75% |
| Wen et al. (2021) | 1 | 1 | 1 | 0 | 1 | 1 | 1 | 1 | 1 | 1 | 1 | 1 | 1 | 1 | 1 | 1 | 0 | 0 | 0 | 1 | 0 | 0 | 1 | 1 | 0 | 1 | 1 | 1 | 75% |
| Zhang et al. (2015) | 0 | 1 | 1 | 0 | 1 | 1 | 1 | 1 | 0 | 1 | 1 | 0 | 1 | 1 | 0 | 1 | 1 | 1 | 0 | 1 | 0 | 1 | 1 | 1 | 0 | 1 | 0 | 1 | 68% |
| Zhang et al. (2016) | 1 | 1 | 1 | 0 | 1 | 1 | 1 | 1 | 1 | 0 | 1 | 0 | 1 | 1 | 0 | 1 | 0 | 0 | 0 | 1 | 0 | 0 | 1 | 1 | 0 | 1 | 0 | 1 | 61% |
| Zhang et al. (2021) | 1 | 1 | 1 | 0 | 1 | 1 | 1 | 1 | 1 | 1 | 1 | 0 | 1 | 1 | 1 | 0 | 1 | 1 | 0 | 1 | 0 | 1 | 1 | 1 | 0 | 1 | 1 | 1 | 79% |
| Zhao et al. (2022) | 1 | 1 | 1 | 0 | 1 | 1 | 1 | 1 | 1 | 1 | 1 | 0 | 1 | 1 | 1 | 1 | 1 | 0 | 0 | 1 | 0 | 1 | 1 | 1 | 0 | 1 | 1 | 1 | 79% |
| Zheng et al. (2020) | 0 | 1 | 1 | 0 | 1 | 1 | 1 | 1 | 1 | 1 | 1 | 0 | 1 | 1 | 0 | 1 | 0 | 0 | 0 | 1 | 0 | 0 | 1 | 1 | 0 | 1 | 1 | 1 | 64% |
| Zhou et al. (2022) | 1 | 1 | 1 | 0 | 1 | 1 | 1 | 1 | 1 | 1 | 1 | 0 | 1 | 1 | 1 | 1 | 1 | 0 | 0 | 1 | 0 | 1 | 1 | 1 | 0 | 1 | 1 | 1 | 79% |
| Zhou et al. (2022) | 1 | 1 | 1 | 0 | 1 | 1 | 1 | 1 | 1 | 1 | 1 | 0 | 1 | 1 | 1 | 1 | 1 | 0 | 0 | 1 | 0 | 1 | 1 | 1 | 0 | 1 | 1 | 1 | 79% |

1, item was appropriately reported; 0, item was not appropriately reported; -, item was not applicable.

**S1D Table. Economic evaluation results of included full-text publications.**

| **Study** | **Location** | **Base case population** | **Study design** | **Treatment line of focus** | **Interventions compared** | **ICER/CER** | **WTP threshold** | **Authors' conclusion** |
| --- | --- | --- | --- | --- | --- | --- | --- | --- |
| Cabibbo et al. (2020) [1] | - | Advanced HCC | Risk-benefit analysis | Sequences | Lenvatinib-nivolumab vs.  lenvatinib-pembrolizumab vs.  atezolizumab plus bevacizumab-nivolumab vs.  sorafenib-nivolumab vs.  atezolizumab plus bevacizumab-pembrolizumab vs.  lenvatinib-ramucirumab vs.  lenvatinib-regorafenib vs.  lenvatinib-cabozantinib vs.  sorafenib-pembrolizumab vs.  atezolizumab plus bevacizumab-ramucirumab vs.  atezolizumab plus bevacizumab-regorafenib vs.  atezolizumab plus bevacizumab-cabozantinib vs.  sorafenib-cabozantinib vs.  sorafenib-regorafenib vs.  sorafenib-ramucirumab | Lenvatinib-nivolumab was favored in 52% of cases, compared to atezolizumab plus bevacizumab-nivolumab (assessed by ISER) | - | Lenvatinib-nivolumab was the most effective sequence, while atezolizumab plus bevacizumab-nivolumab was the safest sequence |
| Cabibbo et al. (2021) [2] | - | Advanced HCC | Risk-benefit analysis | Sequences | Atezolizumab plus bevacizumab-lenvatinib vs.  atezolizumab plus bevacizumab-sorafenib after 2018 vs.  atezolizumab plus bevacizumab-cabozantinib vs.  atezolizumab plus bevacizumab-regorafenib vs.  atezolizumab plus bevacizumab-sorafenib before 2018 vs.  atezolizumab plus bevacizumab-ramucirumab | At a willingness-to-risk threshold of 10% of SAEs for LYG, atezolizumab plus bevacizumab-sorafenib was favored in 72% of cases, while at a threshold of 30% of SAEs for LYG, atezolizumab plus bevacizumab-lenvatinib was favored in 69% of cases (assessed by ISER) | - | Atezolizumab plus bevacizumab-lenvatinib or atezolizumab plus bevacizumab-sorafenib was the most effective sequence, while atezolizumab plus bevacizumab-sorafenib was the safest sequence |
| Cai et al. (2020) [3] | China | Untreated, nonresected advanced HCC | Cost-utility analysis | First line | Lenvatinib vs.  sorafenib | US$11,825.94/QALY gained (patients weighing < 60 kg); US$28,627.12/QALY gained (patients weighing ≥ 60 kg) | 3 times the GDP per capita in 2018 for China | Lenvatinib was cost-effective |
| Camma et al. (2013) [4] | Italy | Advanced HCC and intermediate HCC not eligible to or failed ablative therapies | Cost-utility analysis | First line | Full-dose sorafenib vs.  BSC  Dose-adjusted sorafenib vs.  BSC | €69,344/QALY gained (full-dose sorafenib vs. BSC, > WTP threshold); €34,534/QALY gained (dose-adjusted sorafenib vs. BSC) | €38,000/QALY in 2012 | Dose-adjusted sorafenib was cost-effective |
| Carr et al. (2010) [5] | US | Treatment-naïve advanced HCC | Cost-effectiveness analysis | First line | Sorafenib vs.  BSC | US$62,473/LY gained | US$100,000/LY in 2007 | Sorafenib was cost-effective |
| Chiang et al. (2021) [6] | US | Advanced HCC previously treated with sorafenib | Cost-utility analysis | Second line | Pembrolizumab vs.  placebo | US$340,409/QALY gained (> WTP threshold) | US$150,000/ QALY in 2020 | Pembrolizumab was not cost-effective |
| Chiang et al. (2021) [7] | US | Unresectable HCC | Cost-utility analysis | First line | Atezolizumab plus bevacizumab vs.  sorafenib | US$179,729/QALY gained (> WTP threshold) | US$100,000 and 150,000/QALY in 2020 | Atezolizumab plus bevacizumab was not cost-effective |
| Elsisi et al. (2018) [8] | Egypt | Advanced HCC | Cost-utility analysis | First line | Sorafenib vs.  BSC | US$286,776/QALY gained (> WTP threshold) | 3 times the GDP per capita in 2017 for Egypt | Sorafenib was not cost-effective |
| Guan et al. (2022) [9] | China | Advanced HCC | Cost-utility analysis | First line | Donafenib vs.  sorafenib vs.  lenvatinib | Donafenib was dominant | 3 times the GDP per capita in 2020 for China | Donafenib was cost-effective |
| Gupta et al. (2018) [10] | India | Advanced HCC | Cost-utility analysis | First line | Sorafenib vs.  BSC | US$7,861/QALY gained (> WTP threshold) | 1-3 times the GDP per capita in 2017 for India | Sorafenib was not cost-effective |
| Ho et al. (2018) [11] | Taiwan | Advanced HCC | Cost-utility analysis | First line | Sorafenib combination therapy vs.  sorafenib monotherapy | NT$2,725,943/QALY gained (> WTP threshold) | NT$2,133,930/ QALY in 2014 | Sorafenib combination therapy was not cost-effective |
| Hou et al. (2020) [12] | China | Advanced metastatic or unresectable HCC | Cost-utility analysis | First line | Atezolizumab plus bevacizumab vs.  sorafenib | US$61,613/QALY gained (> WTP threshold) | 3 times the GDP per capita in 2019 for China | Atezolizumab plus bevacizumab was not cost-effective |
| Ikeda et al. (2021) [13] | Japan | Unresectable HCC | Cost-utility analysis | First line | Lenvatinib vs.  sorafenib | Dominant | 7,500,000 JPY/QALY in 2018 | Lenvatinib was cost-effective |
| Kim et al. (2019) [14] | Canada | Unresectable HCC | Cost-utility analysis | First line | Lenvatinib vs.  sorafenib | Dominant | Can$50,000/ QALY in 2018 | Lenvatinib was cost-saving |
| Kobayashi et al. (2019) [15] | Japan | Intermediate and advanced unresectable HCC | Cost-utility analysis | First line | Lenvatinib vs.  sorafenib | Dominant | 5,000,000 JPY/QALY in 2017 | Lenvatinib was cost-effective |
| Li et al. (2021) [16] | China | Advanced HCC with portal vein invasion | Cost-utility analysis | First line | HAIC plus sorafenib vs.  sorafenib | US$77,132/QALY gained (> WTP threshold) | 3 times the GDP per capita in 2019 for China | HAIC plus sorafenib was not cost-effective |
| Li et al. (2022) [17] | China | Advanced or unresectable HCC | Cost-utility analysis | First line | Sintilimab plus bevacizumab biosimilar vs.  sorafenib  Atezolizumab plus bevacizumab vs.  sorafenib | US$39,766.86/QALY gained (sintilimab plus bevacizumab biosimilar vs. sorafenib); US$103,037.66/QALY gained (atezolizumab plus bevacizumab vs. sorafenib, > WTP threshold) | 3 times the GDP per capita in 2020 for China | Sintilimab plus bevacizumab biosimilar was cost-effective if sintilimab patient assistance program was considered; Atezolizumab plus bevacizumab was not cost-effective whether atezolizumab patient assistance program was considered or not |
| Li et al. (2022) [18] | US | Advanced or unresectable HCC | Cost-utility analysis | First line | Atezolizumab plus bevacizumab vs.  nivolumab | US$113,892/QALY gained (healthcare system’s perspective); US$136,584/QALY gained (societal perspective) | US$150,000/ QALY in 2021 | Atezolizumab plus bevacizumab was cost-effective |
| Liao et al. (2019) [19] | US, UK and China | Sorafenib‐resistant HCC | Cost-utility analysis | Second line | Cabozantinib vs.  BSC | US$833,497/QALY gained in the US; US$304,177/QALY gained in the UK; US$156,437/QALY gained in China (all > WTP thresholds) | US$150,000/ QALY for the US, US$70,671/  QALY for the UK, and US$26,481/ QALY (3 times the GDP per capita) for China in 2017 | Cabozantinib was not cost-effective |
| Liu et al. (2022) [20] | China and US | Locally advanced, metastatic or unresectable HCC | Cost-utility analysis | First line | Atezolizumab plus bevacizumab vs.  sorafenib | US$138,462/QALY gained (Markov model) and US$137,056/QALY gained (partitioned survival model) in China; US$492,241/QALY gained (Markov model) and US$485,804/QALY gained (partitioned survival model) in US (all > WTP thresholds) | US$31,499/ QALY (3 times the GDP per capita) for China and US$150,000/ QALY for the US in 2020 | Atezolizumab plus bevacizumab was not cost-effective |
| Meng et al. (2021) [21] | China | Advanced HCC previously treated with sorafenib | Cost-utility analysis | Second line | Pembrolizumab vs.  placebo | ¥1,266,846.18/QALY gained (partitioned survival model); ¥1,288,151.64/QALY gained (Markov model; both > WTP threshold) | 1-3 times the GDP per capita in 2020 for China | Pembrolizumab was not cost-effective |
| Meng et al. (2022) [22] | China | Unresectable or metastatic HCC | Cost-utility analysis | First line | Donafenib vs.  sorafenib | US$41,081.52/QALY gained (> WTP threshold) | 1-3 times the GDP per capita in 2020 for China | Donafenib was not cost-effective |
| Meng et al. (2022) [23] | China | Unresectable or metastatic HCC | Cost-utility analysis | First line | Donafenib vs.  lenvatinib | US$10,790.18/QALY gained | 1-3 times the GDP per capita in 2020 for China | Donafenib was cost-effective |
| Meyers et al. (2021) [24] | Canada | Untreated advanced or unresectable HCC | Cost-utility analysis | First line | Lenvatinib vs.  sorafenib | Dominant | Can$50,000/ QALY in 2019 | Lenvatinib was cost-effective |
| Muszbek et al. (2008) [25] | Canada | Treatment-naïve advanced HCC | Cost-effectiveness analysis | First line | Sorafenib vs.  BSC | Can$75,759/LY gained | Can$100,000/ LY in 2007 | Sorafenib was cost-effective |
| Parikh et al. (2017) [26] | US | Advanced HCC previously treated with sorafenib | Cost-utility analysis | Second line | Regorafenib vs.  BSC | US$224,362/QALY gained (> WTP threshold) | US$100,000/ QALY in 2016 | Regorafenib was not cost-effective |
| Peng et al. (2022) [27] | China | Unresectable HCC | Cost-utility analysis | First line | Sintilimab plus bevacizumab biosimilar vs.  sorafenib | US$23,352/QALY gained | 3 times the GDP per capita in 2020 for China | Sintilimab plus bevacizumab biosimilar was cost-effective |
| Qin et al. (2018) [28] | China | Advanced or metastatic HCC | Cost-utility analysis | First line | FOLFOX4 vs.  sorafenib | Dominant | 3 times the GDP per capita in 2014 for China | FOLFOX4 was dominant |
| Saiyed et al. (2020) [29] | Australia | Treatment-naïve advanced HCC | Cost-utility analysis | First line | Lenvatinib vs.  sorafenib | A$33,028/QALY gained | A$50,000/ QALY in 2020 | Lenvatinib was cost-effective |
| Sangmala et al. (2018) [30] | Thailand | Advanced HCC | Cost-utility analysis | First line | Sorafenib vs.  palliative care | Inferior | 160,000 THB/QALY in 2017 | Palliative care was dominant |
| Sherrow et al. (2020) [31] | US | Advanced HCC | Cost-utility analysis | Sequences | Sorafenib-regorafenib vs.  sorafenib-cabozantinib vs.  sorafenib-pembrolizumab vs.  sorafenib-nivolumab vs.  lenvatinib-regorafenib vs.  lenvatinib-cabozantinib vs.  lenvatinib-pembrolizumab vs.  lenvatinib-nivolumab | Sorafenib-pembrolizumab: US$227,741.03/QALY gained (the lowest CER) | US$300,000 /QALY in 2019 | 1^st^ line TKI therapy-2^nd^ line immunotherapy was the most cost-effective strategy |
| Shi et al. (2021) [32] | China | Advanced HCC previously treated with sorafenib or systemic chemotherapy | Cost-utility analysis | Second line | Two-week regimen of camrelizumab vs.  three-week regimen of camrelizumab | ¥63,703.72/QALY gained | 3 times the GDP per capita in 2019 for China | Two-week regimen of camrelizumab was cost-effective |
| Shlomai et al. (2018) [33] | US | Advanced HCC previously treated with sorafenib | Cost-utility analysis | Second line | Regorafenib vs.  BSC | US$201,797-US$268,506/QALY gained (> WTP threshold) | US$50,000-150,000/QALY in 2017 | Regorafenib was not cost-effective |
| Shlomai et al. (2019) [34] | US | Advanced HCC | Cost-utility analysis | Second line | Cabozantinib vs.  BSC | US$469,374/QALY gained (> WTP threshold) | US$50,000-150,000/QALY in 2018 | Cabozantinib was not cost-effective |
| Sieg et al. (2020) [35] | Germany and US | Advanced HCC received prior sorafenib | Cost-utility analysis | Second line | Cabozantinib vs.  BSC | US$375,470/QALY gained in Germany; US$1,189,706/QALY gained in the US (both > WTP thresholds) | 3 times the GDP per capita in 2018 for Germany and the US | Cabozantinib was not cost-effective |
| Soto-Perez-de-Celis et al. (2019) [36] | US | Advanced HCC received prior sorafenib | Cost-utility analysis | Second line | Cabozantinib vs.  BSC | US$1,040,675/QALY gained (> WTP threshold) | US$100,000/ QALY in 2018 | Cabozantinib was not cost-effective |
| Su et al. (2021) [37] | US | Unresectable HCC | Cost-utility analysis | First line | Atezolizumab plus bevacizumab vs.  sorafenib | US$169,223/QALY gained (> WTP threshold) | US$150,000/ QALY in 2019 | Atezolizumab plus bevacizumab was not cost-effective |
| Wen et al. (2021) [38] | China and US | Unresectable HCC | Cost-utility analysis | First line | Atezolizumab plus bevacizumab vs.  sorafenib | US$145,546.21/QALY gained in China; US$168,030.21/QALY gained in the US (both > WTP thresholds) | 3 times the GDP per capita in 2019 for China and the US | Atezolizumab plus bevacizumab was not cost-effective |
| Zhang et al. (2015) [39] | China | Advanced HCC | Cost-utility analysis | First line | Sorafenib vs.  BSC | US$101,399.11/QALY gained (> WTP threshold) | 3 times the GDP per capita in 2012 for China | Sorafenib was not cost-effective |
| Zhang et al. (2016) [40] | China | Advanced HCC | Cost-utility analysis | First line | FOLFOX4 vs.  sorafenib | US$934,801.57/QALY gained (societal perspective); US$333,966.14/QALY gained (patients’ perspective) | 3 times the GDP per capita in 2014 for China | FOLFOX4 was cost-effective |
| Zhang et al. (2021) [41] | US | Locally advanced metastatic or unresectable HCC | Cost-utility analysis | First line | Atezolizumab plus bevacizumab vs.  sorafenib | US$322,500/QALY gained | US$100,000-150,000/QALY in 2020 | Atezolizumab plus bevacizumab was not cost-effective |
| Zhao et al. (2022) [42] | China | Unresectable HCC | Cost-utility analysis | First line | Sorafenib vs.  lenvatinib vs.  donafenib vs.  sintilimab plus bevacizumab biosimilar vs.  atezolizumab plus bevacizumab | US$40,667.92/QALY gained (lenvatinib, > WTP threshold); US$27,630.63/QALY gained (donafenib); US$51,877.36/QALY gained (sintilimab plus bevacizumab biosimilar, > WTP threshold); US$130,508.44/QALY gained (atezolizumab plus bevacizumab, > WTP threshold, all compared with sorafenib) | 3 times the GDP per capita in 2020 for China | Donafenib was cost-effective (the ranking of cost- effectiveness was as follows: donafenib > sorafenib > lenvatinib > sintilimab plus bevacizumab biosimilar > atezolizumab plus bevacizumab) |
| Zheng et al. (2020) [43] | US | Advanced HCC received prior sorafenib with α-fetoprotein concentrations of at least 400 ng/ml | Cost-utility analysis | Second line | Ramucirumab vs.  placebo | US$782,104.57/QALY gained (> WTP threshold) | US$100,000/ QALY in 2019 | Ramucirumab was not cost-effective |
| Zhou et al. (2022) [44] | China | Unresectable or metastatic HCC | Cost-utility analysis | First line | Sintilimab plus bevacizumab biosimilar vs.  sorafenib | US$20,968/QALY gained | 3 times the GDP per capita in 2020 for China | Sintilimab plus bevacizumab biosimilar was cost-effective |
| Zhou et al. (2022) [45] | China | Unresectable or metastatic HCC | Cost-utility analysis | First line | Sintilimab plus bevacizumab biosimilar vs.  lenvatinib | US$24,462/QALY gained | 1-3 times the GDP per capita in 2021 for China | Sintilimab plus bevacizumab biosimilar was cost-effective |

Abbreviations: BSC, best supportive care; CER, cost-effectiveness ratio; GDP, gross domestic product; HAIC, hepatic arterial infusion of chemotherapy; HCC, hepatocellular carcinoma; ICER, incremental cost-effectiveness ratio; ISER, incremental safety-effectiveness ratio; LYG, life-year gained; QALY, quality-adjusted life-year; SAE, severe adverse event; TKI, tyrosine kinase inhibitor; UK, United Kingdom; US, United States; WTP, willingness-to-pay.

The grey portion in the table was presented in the main text.

**S1E Table. Economic evaluation results of included technology appraisals.**

| **TA** | **Location** | **Base case population** | **Study design** | **Treatment line of focus** | **Interventions compared** | **ICER** | **WTP threshold** | **Authors' conclusion** |
| --- | --- | --- | --- | --- | --- | --- | --- | --- |
| NICE TA 474 (2017) [46] | UK | Advanced HCC when surgical or locoregional therapies had failed or were not suitable | Cost-utility analysis | First line | Sorafenib vs.  BSC | < £50,000/QALY gained | £50,000/QALY | Sorafenib was cost-effective |
| NICE TA 551 (2018) [47] | UK | Untreated, advanced, unresectable HCC | Cost-utility analysis | First line | Lenvatinib vs.  sorafenib | Within the range normally considered to be an acceptable use of NHS resources | - | Lenvatinib was cost-effective |
| NICE TA 555 (2019) [48] | UK | Advanced unresectable HCC previously treated with sorafenib | Cost-utility analysis | Second line | Regorafenib vs.  BSC | < £50,000/QALY gained | £50,000/QALY | Regorafenib was cost-effective |
| NICE TA 666 (2020) [49] | UK | Advanced or unresectable HCC | Cost-utility analysis | First line | Atezolizumab plus bevacizumab vs.  sorafenib  Atezolizumab plus bevacizumab vs.  lenvatinib | < £50,000/QALY gained | £50,000/QALY | Atezolizumab plus bevacizumab was cost-effective |
| NICE TA 849 (2022) [50] | UK | Advanced HCC previously treated with sorafenib | Cost-utility analysis | Second line | Cabozantinib vs.  regorafenib | Within the range normally considered to be an acceptable use of NHS resources | - | Cabozantinib was cost-effective |
| pCODR 10119 (2018) [51] | Canada | Unresectable HCC following treatment with sorafenib | Cost-utility analysis | Second line | Regorafenib vs.  BSC | Between Can$152,657/QALY gained and Can$175,700/QALY gained (> WTP threshold) | - | Regorafenib was not cost-effective |
| pCODR 10134 (2018) [52] | Canada | Sorafenib-refractory or intolerant advanced (not amenable to curative therapy or local therapeutic measures) or metastatic HCC | Cost-utility analysis | Second line | Nivolumab vs.  BSC | Between Can$193,458/QALY gained and unknown (> WTP threshold) | - | Nivolumab was not cost-effective |
| pCODR 10175 (2019) [53] | Canada | Advanced, unresectable HCC | Cost-utility analysis | First line | Lenvatinib vs.  sorafenib | Dominant | - | Lenvatinib was dominant |
| pCODR 10186 (2020) [54] | Canada | Advanced HCC previously treated with sorafenib | Cost-utility analysis | Second line and third line | Cabozantinib vs.  BSC  Cabozantinib vs.  regorafenib | Between Can$285,931/QALY gained and Can$442,810/QALY gained (cabozantinib vs. BSC); Between Can$250,053/QALY gained and Can$320,500/QALY gained (cabozantinib vs. regorafenib, both > WTP threshold) | - | Cabozantinib was not cost-effective |
| pCODR 10217 (2020) [55] | Canada | Unresectable or metastatic HCC | Cost-utility analysis | First line | Atezolizumab plus bevacizumab vs.  sorafenib  Atezolizumab plus bevacizumab vs.  lenvatinib | Can$771,970/QALY gained (atezolizumab plus bevacizumab vs. sorafenib, > WTP threshold) | Can$50,000/ QALY | Atezolizumab plus bevacizumab was not cost-effective |

Abbreviations: BSC, best supportive care; HCC, hepatocellular carcinoma; ICER, incremental cost-effectiveness ratio; NHS, National Health Service; NICE, National Institute for Health and Care Excellence; pCODR, pan-Canadian Oncology Drug Review; QALY, quality-adjusted life-year; TA, technology appraisal; UK, United Kingdom; WTP, willingness-to-pay.

The grey portion in the table was presented in the main text.

**S1F Table. Summary of clinical effectiveness inputs.**

| **Study** | **Sources of evidence** | **Time-to-event distributions** | | **Methods of HCC progression** |
| --- | --- | --- | --- | --- |
|  |  | **PFS** | **OS** |  |
| Cabibbo et al. (2020) [1] | RCT aggregate (REFLECT, IMbrave 150, RESORCE, CELESTIAL, Asian cohort of CheckMate 040, KEYNOTE-240, SUN1170, SIRveNIB, SILIUS, SARAH, REACH) | Weibull | Weibull | TPs |
| Cabibbo et al. (2021) [2] | RCT aggregate (IMbrave 150, SHARP, NCT00492752, BRISK-FL, SUN1170, NCT01009593, SEARCH, REFLECT, RESORCE, CELESTIAL, REACH-2, REACH) | Weibull | Weibull | TPs |
| Cai et al. (2020) [3] | RCT aggregate (Chinese cohort of REFLECT) | Weibull | Weibull | TPs  Approach used to estimate TPs: survival modelling  $TP (t)= 1 - exp[\lambda\left( t-u \right)^{\gamma}-\lambda t^{\gamma}]$  λ = scale parameter, γ = shape parameter, u = cycle length |
| Camma et al. (2013) [4] | RWD IPD (SOFIA, prospective) | Weibull | Weibull | TPs  Approach used to estimate TPs: survival modelling |
| Carr et al. (2010) [5] | RCT IPD (SHARP) | Lognormal | Lognormal | TPs |
| Chiang et al. (2021) [6] | RCT aggregate (KEYNOTE-240) | Weibull | Weibull | TPs |
| Chiang et al. (2021) [7] | RCT aggregate (IMbrave 150) | Weibull | Weibull | TPs |
| Elsisi et al. (2018) [8] | RWD IPD (retrospective) | - | - | TPs  Sorafenib:  PFS-PD: 0.157 (0.125-0.188)  PFS-death: 0.002 (0.002-0.003)  PD-death: 0.500 (0.400-0.600)  BSC:  PFS-PD: 0.290 (0.232-0.349)  PFS-death: 0.002 (0.002-0.003)  PD-death: 0.219 (0.175-0.263)  Distribution: gamma |
| Guan et al. (2022) [9] | RCT aggregate (network meta-analysis of ZGDH3 and REFLECT) | Donafenib: lognormal  Lenvatinib: based on HR of lenvatinib vs. donafenib: 0.73  Sorafenib: based on HR of sorafenib vs. donafenib: 1.10  Distribution: lognormal | Donafenib: lognormal  Lenvatinib: based on HR of lenvatinib vs. donafenib: 1.11  Sorafenib: based on HR of sorafenib vs. donafenib: 1.20  Distribution: lognormal | Survival functions |
| Gupta et al. (2018) [10] | RCT aggregate (NCT00492752) | - | - | TPs  Sorafenib:  PFS-PD: 0.179 (95% CI: 0.158-0.199)  PFS-death: 0.004 (95% CI: 0.003-0.004)  PD-death: 0.375 (95% CI: 0.333-0.417)  BSC:  PFS-PD: 0.357 (95% CI: 0.317-0.398)  PFS-death: 0.004 (95% CI: 0.003-0.004)  PD-death: 0.412 (95% CI: 0.262-0.328)  Distribution: beta |
| Ho et al. (2018) [11] | RWD IPD (retrospective) | - | - | TPs  Approach used to estimate TPs: survival modelling  $T{P (1 month)= 1 - (0.5)}^{(1/{median} time to event)}$  derived from:  ${TP= 1 - e}^{- R}$, $R=- ln [0.5]/(time to event/number of treatment cycles)$  Sorafenib monotherapy:  PFS-PD: 0.2264  PFS-death: 0.1097  PD-death: 0.1912  Sorafenib combination therapy:  PFS-PD: 0.1399  PFS-death: 0.0739  PD-death: 0.1447  Distribution: beta |
| Hou et al. (2020) [12] | RCT aggregate (IMbrave150) | Atezolizumab-bevacizumab: Royston-Parmar spline  Sorafenib: lognormal | Atezolizumab-bevacizumab: lognormal  Sorafenib: log-logistic | Survival functions |
| Ikeda et al. (2021) [13] | RCT IPD (Japanese cohort of REFLECT) | Lognormal | Log-logistic | Survival functions |
| Kim et al. (2019) [14] | RCT aggregate (REFLECT) | Lenvatinib: log-logistic  Sorafenib: lognormal | Lenvatinib: log-logistic  Sorafenib: lognormal | TPs  Approach used to estimate TPs: survival modelling |
| Kobayashi et al. (2019) [15] | RCT IPD (REFLECT) | Lognormal | Log-logistic | Survival functions |
| Li et al. (2021) [16] | RCT aggregate (NCT02774187) | Weibull | Weibull | TPs  Approach used to estimate TPs: survival modelling |
| Li et al. (2022) [17] | RCT aggregate (network meta-analysis of ORIENT-32 and IMbrave150) | Sorafenib: lognormal  Sintilimab-bevacizumab biosimilar: based on HR of sintilimab-bevacizumab biosimilar vs. sorafenib: 0.570 (0.47-0.70)  Atezolizumab-bevacizumab: based on HR of atezolizumab-bevacizumab vs. sorafenib: 0.600 (0.47-0.76)  Distribution: lognormal | Sorafenib: log-logistic  Sintilimab-bevacizumab biosimilar: based on HR of sintilimab-bevacizumab biosimilar vs. sorafenib: 0.570 (0.43-0.75)  Atezolizumab-bevacizumab: based on HR of atezolizumab-bevacizumab vs. sorafenib: 0.580 (0.42-0.79)  Distribution: lognormal | Survival functions |
| Li et al. (2022) [18] | RCT aggregate (network meta-analysis of IMbrave150 and CheckMate 459) | Lognormal | Lognormal | Survival functions |
| Liao et al. (2019) [19] | RCT aggregate (CELESTIAL) | - | - | TPs  Approach used to estimate TPs: model calibration  Cabozantinib:  PFS-PD: 0.091 (0.0637‐0.1183)  PFS-death: 0.054 (0.0378‐0.0702)  PD-death: 0.083 (0.0581‐0.1079)  BSC:  PFS-PD: 0.218 (0.1526‐0.2834)  PFS-death: 0.082 (0.0574‐0.1066)  PD-death: 0.093 (0.0651‐0.1209) |
| Liu et al. (2022) [20] | RCT aggregate (IMbrave 150, Chinese cohort of IMbrave 150) | China:  Lognormal  US:  Atezolizumab-bevacizumab: exponential  Sorafenib: log-logistic | China:  Log-logistic  US:  Lognormal | TPs and survival functions  Approach used to estimate TPs: survival modelling |
| Meng et al. (2021) [21] | RCT aggregate (KEYNOTE-240) | Log-logistic | Lognormal | TPs and survival functions  Approach used to estimate TPs: survival modelling |
| Meng et al. (2022) [22] | RCT aggregate (ZGDH3) | Donafenib: lognormal  Sorafenib: log-logistic | Lognormal | Survival functions |
| Meng et al. (2022) [23] | RCT aggregate (network meta-analysis of ZGDH3 and REFLECT) | Lognormal | Lognormal | Survival functions |
| Meyers et al. (2021) [24] | RCT aggregate (REFLECT) | Lognormal | Log-logistic | Survival functions |
| Muszbek et al. (2008) [25] | RCT IPD (SHARP) | Lognormal | Lognormal | TPs |
| NICE TA 474 (2017) [46] | RCT IPD (SHARP) | Lognormal (treatment duration) | 75% lognormal and 25% Weibull | TPs  Approach used to estimate TPs: survival modelling |
| NICE TA 551 (2018) [47] | RCT IPD (REFLECT) | Generalised gamma | Log-logistic | Survival functions |
| NICE TA 555 (2019) [48] | RCT IPD (RESORCE) | - | Weibull | Survival functions |
| NICE TA 666 (2020) [49] | RCT IPD (IMbrave 150, network meta-analysis of IMbrave 150, REFLECT, and CheckMate 459) | Lognormal | Lognormal | Survival functions |
| NICE TA 849 (2022) [50] | RCT IPD and aggregate data (CELESTIAL, RESORCE) | - | - | Survival functions |
| Parikh et al. (2017) [26] | RCT aggregate (RESORCE) | - | - | TPs  Assumed constant HCC progression rates over time |
| pCODR 10119 (2018) [51] | RCT IPD (RESORCE) | Lognormal | Lognormal | Survival functions |
| pCODR 10134 (2018) [52] | RCT IPD (CheckMate 040, RESORCE) | Generalised gamma  (Gompertz for time to discontinuation curve) | Log-logistic | Survival functions |
| pCODR 10175 (2019) [53] | RCT IPD (REFLECT) | - | - | Survival functions |
| pCODR 10186 (2020) [54] | RCT IPD and aggregate data (CELESTIAL, RESORCE) | - | Cabozantinib vs. BSC: generalised gamma  Cabozantinib vs. regorafenib: log-logistic | Survival functions |
| pCODR 10217 (2020) [55] | RCT IPD (IMbrave 150, network meta-analysis of IMbrave 150, REFLECT, and CheckMate 459) | - | - | Survival functions |
| Peng et al. (2022) [27] | RCT aggregate (ORIENT-32) | Sorafenib: lognormal  Sintilimab-bevacizumab biosimilar: based on HR of sintilimab-bevacizumab biosimilar vs. sorafenib: 0.560 (0.460-0.700)  Distribution: lognormal | Sorafenib: log-logistic  Sintilimab-bevacizumab biosimilar: based on HR of sintilimab-bevacizumab biosimilar vs. sorafenib: 0.570 (0.430-0.750)  Distribution: lognormal | TPs |
| Qin et al. (2018) [28] | RCT aggregate (ORIENTAL, EACH) | - | - | TPs |
| Saiyed et al. (2020) [29] | RCT aggregate (REFLECT) | Lenvatinib: lognormal  Sorafenib: log-logistic | Lenvatinib: lognormal  Sorafenib: log-logistic | Survival functions |
| Sangmala et al. (2018) [30] | RWD IPD (retrospective) | - | - | TPs  Approach used to estimate TPs: non-parametric count method  Sorafenib:  1^st^ line no progression-1^st^ line no progression: 0.4227 ± 0.0309  1^st^ line no progression-1^st^ line continued post progression: 0.2088 ± 0.0203  1^st^ line no progression-palliative care: 0.1955 ± 0.0198  1^st^ line no progression-death: 0.1731  1^st^ line continued post progression-1^st^ line continued post progression: 0.4451  1^st^ line continued post progression-palliative care: 0.4152 ± 0.0616  1^st^ line continued post progression-death: 0.5848  Palliative care-palliative care: 0.4152 ± 0.0616  Palliative care-death: 0.1397 ± 0.0096  Palliative care:  1^st^ line no progression-1^st^ line no progression: 0.7330  1^st^ line no progression-palliative care post-progression: 0.1520 ± 0.0026  1^st^ line no progression-death: 0.1150 ± 0.0045  Palliative care post-progression-palliative care post-progression: 0.7808  Palliative care post-progression-death: 0.2192 ± 0.0030 |
| Sherrow et al. (2020) [31] | RCT aggregate (SHARP, REFLECT, RESORCE, CELESTIAL, KEYNOTE-224, CheckMate 040) | - | - | TPs |
| Shi et al. (2021) [32] | RCT aggregate (NCT02989922) | Log-logistic | Two-week regimen: exponential  Three-week regimen: lognormal | Survival functions |
| Shlomai et al. (2018) [33] | RCT aggregate (RESORCE) | Weibull | Weibull | TPs  Approach used to estimate TPs: survival modelling |
| Shlomai et al. (2019) [34] | RCT aggregate (CELESTIAL) | Weibull | Weibull | TPs  Approach used to estimate TPs: survival modelling |
| Sieg et al. (2020) [35] | RCT aggregate (CELESTIAL) | Weibull | Weibull | TPs  PFS-PFS: $e^{\frac{yPFS}{number of treatment cycles}}$  PFS-death: ${1 - e}^{\frac{yOS}{number of treatment cycles}}$  PD-death: ${1 - e}^{\frac{yOS-yPFS}{number of treatment cycles}}$ |
| Soto-Perez-de-Celis et al. (2019) [36] | RCT aggregate (CELESTIAL) | - | - | - |
| Su et al. (2021) [37] | RCT aggregate (IMbrave 150) | Atezolizumab-bevacizumab: Royston-Parmar spline  Sorafenib: lognormal | Atezolizumab-bevacizumab: lognormal  Sorafenib: log-logistic | Survival functions |
| Wen et al. (2021) [38] | RCT aggregate (IMbrave 150) | - | - | TPs  Approach used to estimate TPs: model calibration  Atezolizumab-bevacizumab:  PFS-PD: 0.0656  PFS-death: 0.0263  PD-death: 0.0495  Sorafenib:  PFS-PD: 0.1159  PFS-death: 0.0398  PD-death: 0.0813 |
| Zhang et al. (2015) [39] | RWD IPD (retrospective) and RCT aggregate (NCT00492752) | - | - | TPs  Approach used to estimate TPs: survival modelling  ${TP (1 month)= 1 - (0.5)}^{(1/{median} time to event)}$  derived from:  ${TP= 1 - e}^{- R}$, $R=- ln [0.5]/(time to event/number of treatment cycles)$  Sorafenib:  PFS-PD: 0.143  PFS-death: 0.083  PD-death: 0.180  BSC:  PFS-PD: 0.390  PFS-death: 0.152  PD-death: 0.219 |
| Zhang et al. (2016) [40] | RCT aggregate (ORIENTAL, EACH) | - | - | TPs  Approach used to estimate TPs: survival modelling  ${TP (1 month)= 1 - (0.5)}^{(1/{median} time to event)}$  derived from:  ${TP= 1 - e}^{- R}$, $R=- ln [0.5]/(time to event/number of treatment cycles)$  FOLFOX4:  PFS-PFS: 0.686  PFS-PD: 0.211  PFS-death: 0.103  PD-PD: 0.819  PD-death: 0.181  Sorafenib:  PFS-PFS: 0.680  PFS-PD: 0.219  PFS-death: 0.101  PD-PD: 0.829  PD-death: 0.171 |
| Zhang et al. (2021) [41] | RCT aggregate (IMbrave 150) | Weibull | Weibull | Survival functions |
| Zhao et al. (2022) [42] | RCT aggregate (network meta-analysis of Chinese cohorts of IMbrave150, REFLECT, ORIENT-32 and ZGDH3) | Sorafenib: lognormal  Lenvatinib, donafenib, sintilimab-bevacizumab biosimilar, and  atezolizumab-bevacizumab: based on HRs of regimens vs. sorafenib | Sorafenib: lognormal  Lenvatinib, donafenib, sintilimab-bevacizumab biosimilar, and  atezolizumab-bevacizumab: based on HRs of regimens vs. sorafenib | Survival functions |
| Zheng et al. (2020) [43] | RCT aggregate (REACH-2) | - | - | TPs  Approach used to estimate TPs: survival modelling  ${TP (1 month)= 1 - (0.5)}^{(1/{median} time to event)}$  derived from:  ${TP= 1 - e}^{- R}$, $R=- ln [0.5]/(time to event/number of treatment cycles)$  Ramucirumab:  PFS-PFS: 0.703  PFS-PD: 0.219  PFS-death: 0.078  PD-PD: 0.885  PD-death: 0.115  Placebo:  PFS-PFS: 0.557  PFS-PD: 0.352  PFS-death: 0.091  PD-PD: 0.885  PD-death: 0.115 |
| Zhou et al. (2022) [44] | RCT IPD (ORIENT-32) | Lognormal | Sintilimab-bevacizumab biosimilar: lognormal  Sorafenib: log-logistic | Survival functions |
| Zhou et al. (2022) [45] | RCT IPD and aggregate data (network meta-analysis of ORIENT-32 and REFLECT) | Lognormal | Lognormal | Survival functions |

Abbreviations: BSC, best supportive care; HCC, hepatocellular carcinoma; HR, hazard ratio; IPD, individual patient data; NICE, National Institute for Health and Care Excellence; OS, overall survival; pCODR, pan-Canadian Oncology Drug Review; PD, progressive disease; PFS, progression-free survival; RCT, randomized controlled trial; RWD, real-world data; TA, technology appraisal; TPs, transition probabilities; US, United States.

The grey portion in the table was presented in the main text.

**S1G Table. Summary of utility and cost inputs.**

| **Study** | **Perspective** | **Utility** | | | | | **Cost scope** |
| --- | --- | --- | --- | --- | --- | --- | --- |
|  |  | **PFS** | **PD** | **AE disutility** | **Distribution** | **Source** |  |
| Cabibbo et al. (2020) [1] | - | - | - | - | - | - | - |
| Cabibbo et al. (2021) [2] | - | - | - | - | - | - | - |
| Cai et al. (2020) [3] | Healthcare system | 0.845 | 0.714 | Diarrhea: 0.014  Asthenic conditions: 0.108  Decreased appetite: 0.078  Abdominal pain: 0.005  Weight decreased: 0.053  Vomiting: 0.047  Hypertension: 0.000  Hand-foot syndrome: 0.016 | - | Published literature | Direct medical costs: drugs, follow-up, severe AE treatment, post-progression of the disease costs |
| Camma et al. (2013) [4] | Payer | 0.76 (0.76-0.80) | 0.68 (0.60-0.68) | - | - | Published literature | Direct medical costs: inpatient and outpatient visits, diagnostic and laboratory testing, medications, procedures costs |
| Carr et al. (2010) [5] | Payer | - | - | - | - | - | Direct medical costs: drugs, physician visits, laboratory tests, scans, hospitalizations, AEs costs |
| Chiang et al. (2021) [6] | Payer | Pembrolizumab: 0.84 (0.79-0.88)  Placebo: 0.76 (0.59-0.93) | 0.68 (0.54-0.82) | Fatigue: 0.11 (0.08-0.14)  Nausea: 0.13 (0.09-0.15)  Hypothyroidism: 0  Pneumonitis: 0.17 (0.11-0.22)  Skin reaction: 0.13 (0.09-0.15)  Hepatitis: 0.17 (0.11-0.22)  Colitis: 0.17 (0.12-0.22)  Hypophysitis: 0.17 (0.11-0.22)  Type 1 DM: 0.17 (0.11-0.22) | Beta | Published literature | Direct medical costs: drugs, drug administration, management of AEs costs |
| Chiang et al. (2021) [7] | Payer | 0.78 (0.624-0.936) | 0.68 (0.54-0.82) | Diarrhea: 0.103 (0.082-0.123)  Hand-foot syndrome: 0.116 (0.093-0.139)  Hypertension: 0  Increased blood bilirubin: 0 | Triangular | EQ-5D data collected in clinical trial, published literature | Direct medical costs: drugs, administration, management of AEs costs |
| Elsisi et al. (2018) [8] | Hospital | 0.760 (0.608-0.912) | 0.680 (0.544-0.816) | - | Beta | Published literature | Direct medical costs: medications, physician visits, hospitalizations, management of AEs, laboratory and radiology tests costs |
| Guan et al. (2022) [9] | Healthcare system | 0.76 (0.61-0.91) | 0.68 (0.54-0.82) | Hand-foot syndrome: 0.02 (0.01-0.02)  Hypertension: 0  Elevated AST: 0  Elevated blood bilirubin: 0  Decreased platelet count: 0.20 (0.16-0.24)  Diarrhea: 0.01 (0.01-0.02)  Rash: 0.10 (0.08-0.12)  Fatigue: 0.07 (0.06-0.08) | Beta | Published literature | Direct medical costs: drug acquisition, disease management, EOL care, management of severe AEs costs |
| Gupta et al. (2018) [10] | Societal | 0.76 (95% CI: 0.67-0.85) | 0.68 (95% CI: 0.60-0.76) | - | Beta | Published literature | Direct medical costs: routine care, diagnostics, management of complications costs in both arms; AEs costs in the sorafenib arm |
| Ho et al. (2018) [11] | Payer | 0.76 (0.53-0.99) | 0.68 (0.48-0.88) | - | Beta | Published literature | Direct medical costs: inpatient and outpatient care, pharmacy visits costs |
| Hou et al. (2020) [12] | Healthcare system | 0.76 (0.61-0.91) | 0.68 (0.54-0.82) | Grade 1-2: 0.01 (0.008-0.020)  Grade ≥ 3: 0.16 (0.110-0.204) | Beta | Published literature | Direct medical costs: drug acquisition, follow-up, management of AEs, post-progression, EOL care costs |
| Ikeda et al. (2021) [13] | Healthcare system | 0.79 | 0.78 | - | - | Adjusted EQ-5D data from REFLECT to reflect Japan-specific utilities | Direct medical costs: primary drug-therapy, medical resource-use, AE treatment, post-progression therapy, EOL costs |
| Kim et al. (2019) [14] | Healthcare system | 0.760 (0.546-1.014) | 0.680 (0.476-0.806) | Hand-foot syndrome: 0.116 (0.093-0.139)  Diarrhea: 0.050 (0.019-0.096)  Hypertension: 0  Decreased appetite: 0  Weight decreased: 0  Fatigue: 0.070 (0.036-0.114)  Proteinuria: 0  Nausea/vomiting: 0.050 (0.019-0.096)  Decreased platelet count: 0  Elevated AST: 0  Increased blood bilirubin: 0 | - | Published literature | Direct medical costs: treatment-emergent AEs, treatment, health state costs |
| Kobayashi et al. (2019) [15] | Payer | 0.845 | 0.714 | Diarrhea: 0.014  Asthenic conditions: 0.108  Decreased appetite: 0.078  Abdominal pain: 0.005  Weight decreased: 0.053  Vomiting: 0.047  Hypertension: 0.000  Hand-foot syndrome: 0.016 | Beta | EQ-5D data collected in clinical trial, published literature | Direct medical costs: primary drug-therapy, medical resource-use, AE treatment, post-progression therapy, EOL costs |
| Li et al. (2021) [16] | Healthcare system | 0.76 (0.61-0.91) | 0.68 (0.54-0.82) | Elevated ALT/AST: 0  Neutropenia: 0.09 (0.059-0.12)  Hand-foot syndrome: 0.016 (0.013-0.019)  Diarrhea: 0.047 (0.016-0.077)  Nausea/vomiting: 0.048 (0.038-0.058) | Beta | Published literature | Direct medical costs: induction and subsequent treatments, examination, hospitalization, hepatic artery catheterization, hepatectomy, treatment for grade 3-4 severe AEs, BSC costs |
| Li et al. (2022) [17] | Healthcare system | 0.76 (0.61-0.91) | 0.68 (0.54-0.82) | Grade ≥ 3: 0.16 (0.13-0.19) | Beta | Published literature | Direct medical costs: drugs, tests, grade 3-4 AEs, follow-up, post-progression costs |
| Li et al. (2022) [18] | Healthcare system and societal | 0.76 (0.57-0.95) | 0.68 (0.54-0.82) | Grade 1-2: 0.01 (0.008-0.012)  Grade ≥ 3: 0.16 (0.12-0.20) | Beta | Published literature | Direct medical costs: acquiring drugs, patient’s health state, supportive care, EOL care, AE-related costs  Indirect costs: patient time and/or salary loss, transportation, caregiver costs |
| Liao et al. (2019) [19] | Payer | 0.76 (0.532-0.988) | 0.68 (0.476-0.884) | - | Beta | Published literature | Direct medical costs: drugs, computed tomography, management of grade 3‐4 AEs costs |
| Liu et al. (2022) [20] | Healthcare system (China) and payer (US) | 0.845 (0.676-1) | 0.714 (0.571-0.857) | Hypertension: 0  Diarrhea: 0.014 (0.011-0.017)  Hand-foot syndrome: 0.016 (0.013-0.019) | Beta | Published literature | Direct medical costs: drugs, infusion, AEs costs |
| Meng et al. (2021) [21] | Healthcare system | Pembrolizumab: 0.84 (0.79-0.88)  Placebo: 0.76 (0.59-0.93) | 0.68 (0.54-0.82) | - | Beta | Published literature | Direct medical costs: drugs, disease management, laboratory tests, AEs costs |
| Meng et al. (2022) [22] | Payer | 0.745 (0.730-0.760) | 0.678 (0.655-0.701) | Hand-foot syndrome: 0.116 (0.093-0.139)  Hypertension: 0.012 (0.010-0.014)  Elevated AST: 0  Hypophosphatemia: 0.181 (0.145-0.217) | Beta | Published literature | Direct medical costs: drug acquisition, administration, follow-up visit, subsequent treatment after disease progression, management of AEs, EOL costs |
| Meng et al. (2022) [23] | Payer | 0.745 (0.730-0.760) | 0.678 (0.655-0.701) | Hand-foot syndrome: 0.116 (0.093-0.139)  Hypertension: 0.012 (0.010-0.014)  Decreased appetite: 0.078 (0.062-0.094)  Weight decreased: 0.053 (0.042-0.064)  Proteinuria: 0.048 (0.005-0.091)  Decreased platelet count: 0.108 (0.086-0.130)  Elevated AST: 0  Increased blood bilirubin: 0 | Beta | Published literature | Direct medical costs: drug acquisition, administration, follow-up visit, subsequent treatment after disease progression, management of AEs, EOL costs |
| Meyers et al. (2021) [24] | Healthcare system | 0.745 | 0.678 | - | - | EQ-5D data collected in clinical trial | Direct medical costs: drug therapy, medical resource use, AE management, EOL costs |
| Muszbek et al. (2008) [25] | Healthcare system | - | - | - | - | - | Direct medical costs: drugs, physician visits, laboratory tests, scans, hospitalizations, AEs costs |
| NICE TA 474 (2017) [46] | Healthcare system | 0.69 | 0.71 | Grade 3-4: 0.012 | Beta | FACT-Hep data collected in clinical trial | Direct medical costs: drugs, hospitalizations, medical staff visits, lab tests, radiological tests, AEs, EOL costs |
| NICE TA 551 (2018) [47] | Healthcare system | 0.745 | 0.678 | - | - | EQ-5D data collected in clinical trial | - |
| NICE TA 555 (2019) [48] | Healthcare system | 0.80-0.82 | 0.75-0.78 | 0-0.03 | - | EQ-5D data collected in clinical trial | Direct medical costs: drugs, hospitalizations, medical staff visits, lab tests, radiological tests, AEs costs |
| NICE TA 666 (2020) [49] | Healthcare system | Time to death utility approach: > 30 weeks, > 15 to ≤ 30 weeks, > 5 to ≤ 15 weeks, and ≤ 5 weeks before death (base case)  (utilities were different across the treatment arms in scenario analyses) | | - | - | EQ-5D data collected in clinical trial | Considered AEs costs |
| NICE TA 849 (2022) [50] | Healthcare system | -  (included age-adjusted utilities) | - | Considered | Beta | EQ-5D data collected in clinical trial | Direct medical costs: drug acquisition, disease management, tests associated with disease progression, management of AEs, EOL costs |
| Parikh et al. (2017) [26] | Healthcare system | 0.76 (0.59- 0.93) | 0.76 (0.59- 0.93) | Hypertension: 0.025 (0.019-0.031)  Hand-foot syndrome: 0.116 (0.088-0.144)  Fatigue: 0.115 (0.087-0.143)  Diarrhea: 0.103 (0.078-0.128) | - | EQ-5D and FACT-Hep data collected in clinical trial | Direct medical costs: drugs, surveillance imaging, AEs, BSC costs |
| pCODR 10119 (2018) [51] | Healthcare system | 0.76 | 0.68 | Considered | - | Published literature | Direct medical costs: drug acquisition, disease management, monitoring, AEs costs |
| pCODR 10134 (2018) [52] | Healthcare system | 0.76 | 0.68 | Considered | - | Published literature | Direct medical costs: drug acquisition, drug administration and monitoring, management of disease progression, EOL care, management of AEs costs |
| pCODR 10175 (2019) [53] | Payer and societal | -  (assumed equivalent utilities across the treatment arms) | - | Considered | - | EQ-5D data collected in clinical trial | Direct medical costs: drug acquisition, health state resource use, AEs, treatment post-progression, death, wastage costs |
| pCODR 10186 (2020) [54] | Payer | - | - | - | - | EQ-5D data collected in clinical trial | - |
| pCODR 10217 (2020) [55] | Payer | - | - | Considered | - | - | - |
| Peng et al. (2022) [27] | Healthcare system | 0.760 (0.610-0.910) | 0.680 (0.540-0.820) | Grade 1-2: 0.010 (0.008-0.020)  Grade ≥ 3: 0.160 (0.110-0.204) | Beta | Published literature | Direct medical costs: drugs, testing, AEs management, best supportive treatment costs |
| Qin et al. (2018) [28] | Healthcare system | 0.76 (0.61-0.91) | 0.68 (0.54-0.82) | - | Normal | Published literature | Direct medical costs: therapies, tests, general ward, treatment of AEs costs |
| Saiyed et al. (2020) [29] | Healthcare system | 0.745 (95% CI: 0.73-0.76) | 0.678 (95% CI: 0.655-0.701) | Diarrhea: 0.05  Fatigue: 0.07  Nausea/vomiting: 0.05  Hypertension: 0.012  Hand-foot syndrome: 0.116 | Beta | Published literature | Direct medical costs: drugs, disease management, AEs, EOL costs |
| Sangmala et al. (2018) [30] | Payer | 0.72 | 0.72 | - | Beta | SF-6D data collected from Chinese HCC patients | Direct medical costs: drugs, disease management, AEs costs |
| Sherrow et al. (2020) [31] | - | 0.84  Patients with toxicity: 0.70 | 0.50 | - | Beta | Published literature | - |
| Shi et al. (2021) [32] | Healthcare system | 0.76 (0.61-0.91) | 0.68 (0.54-0.82) | - | Beta | Published literature | Direct medical costs: drugs, disease management, laboratory tests, AEs costs |
| Shlomai et al. (2018) [33] | Payer | 0.76 (0.61-0.91) | 0.68 (0.54-0.82) | Hand-foot syndrome: 0.116 (0.093-0.139)  Hypertension: 0  Diarrhea: 0.103 (0.082-0.123)  Fatigue: 0.115 (0.093-0.139) | Normal (utilities of base and progression)  Beta (disutilities of AEs) | EQ-5D data collected in clinical trial | Direct medical costs: drugs, disease management, AEs costs |
| Shlomai et al. (2019) [34] | Payer | 0.76 (0.61-0.91) | 0.68 (0.54-0.82) | Hand-foot syndrome: 0.116 (0.093-0.139)  Hypertension: 0  Diarrhea: 0.103 -0.082-0.123)  Fatigue: 0.115 (0.093-0.139) | Normal (utilities of base and progression)  Beta (disutilities of AEs) | EQ-5D data collected in clinical trial | Direct medical costs: drugs, disease management, AEs costs |
| Sieg et al. (2020) [35] | Payer | 0.76 (0.72-0.8)  (utilities were different across the treatment arms in scenario analysis) | 0.68 (0.64-0.72) | - | Normal | Published literature | Direct medical costs: drugs, monitoring, AEs costs |
| Soto-Perez-de-Celis et al. (2019) [36] | Healthcare system | Cabozantinib: 0.817 (95% CI: 0.78-0.86)  Placebo: 0.77 (95% CI: 0.73-0.81) | Cabozantinib: 0.817 (95% CI: 0.78-0.86)  Placebo: 0.77 (95% CI: 0.73-0.81) | Diarrhea: 0.53 (95% CI: 0.48-0.59)  Hand-foot syndrome: 0.47 (95% CI: 0.41-0.52)  Fatigue: 0.59 (95% CI: 0.54-0.64)  Hypertension: 0.64 (95% CI: 0.59-0.69)  Nausea/vomiting: 0.54 (95% CI: 0.48-0.59)  Mucositis: 0.53 (95% CI: 0.47-0.57)  Other grade 3-4 AEs: 0.055 | - | EQ-5D data collected in clinical trial, published literature | Direct medical costs: drugs, surveillance, AEs, EOL costs |
| Su et al. (2021) [37] | Payer | 0.76 (0.61-0.91) | 0.68 (0.54-0.82) | Grade 1-2: 0.01 (0.008-0.020)  Grade ≥ 3: 0.160 (0.110-0.204) | Beta | Published literature | Direct medical costs: drugs, patient’s health state, AEs, EOL costs |
| Wen et al. (2021) [38] | Payer | 0.76 (0.608-0.912) | 0.68 (0.544-0.816) | - | - | Published literature | Direct medical costs: drugs, necessary tests for efficacy and toxicity evaluation in the monitoring period, grade 3-4 AEs-related costs |
| Zhang et al. (2015) [39] | Payer | 0.76 | 0.68 | - | - | Published literature | Direct medical costs: drugs, tests, grade 3-4 AE treatments costs |
| Zhang et al. (2016) [40] | Societal and patient | 0.76 | 0.68 | - | - | Published literature | Direct medical costs: drugs, drug administration, venous access management, nursing care, tests, hospitalization, treatments for grade 3-4 AEs costs;  Indirect costs: time loss costs |
| Zhang et al. (2021) [41] | Payer | 0.837 (0.532-0.988) | 0.714 (0.476-0.884) | - | Beta | Published literature | Direct medical costs: drugs, drug administration, follow-up visits, management of AEs, EOL costs |
| Zhao et al. (2022) [42] | Healthcare system | 0.76 (0.61-0.91) | 0.68 (0.54-0.82) | Grade 1-2: 0.01 (0.01-0.02)  Grade ≥ 3: 0.16 (0.11-0.20) | Beta | Published literature | Direct medical costs: drugs, follow-up, monitoring, EOL care, treatment of grades 3-4 AEs costs |
| Zheng et al. (2020) [43] | Payer | Ramucirumab: 0.778  Placebo: 0.807 | Ramucirumab: 0.688  Placebo: 0.635 | - | - | EQ-5D data collected in clinical trial | Direct medical costs: drugs, necessary tests, therapies for AEs costs |
| Zhou et al. (2022) [44] | Healthcare system | 0.76 (0.53-0.99) | 0.68 (0.48-0.88) | Hypertension: 0.012 (0.010-0.014)  Decreased platelet count: 0.000 (0.000-0.001)  Hand-foot syndrome: 0.116 (0.093-0.134)  Increased blood bilirubin: 0.000 (0.000-0.001)  Increased gamma-glutamyltransferase: 0.000 (0.000-0.001)  Abnormal liver function: 0.160 (0.128-0.192)  Increased conjugated bilirubin: 0.000 (0.000-0.001)  Anaemia: 0.160 (0.128-0.192)  Decreased white blood cell count: 0.000 (0.000-0.001)  Decreased neutrophil count: 0.000 (0.000-0.001)  Decreased lymphocyte count: 0.000 (0.000-0.001)  Ascites: 0.160 (0.128-0.192)  Hepatic failure: 0.160 (0.128-0.192)  Hypokalemia: 0.000 (0.000-0.001)  Hyponatremia: 0.000 (0.000-0.001)  Elevated ALT: 0.000 (0.000-0.001)  Elevated AST: 0.000 (0.000-0.001)  Diarrhea: 0.050 (0.040-0.060) | Beta | Published literature | Direct medical costs: drug use, subsequent treatment, drug administration, management of AEs, follow-up, EOL care costs |
| Zhou et al. (2022) [45] | Healthcare system | 0.745 (0.730-0.760) | 0.678 (0.655-0.701) | Hypertension: 0.12 (0.10-0.14)  Proteinuria: 0.12 (0.10-0.14)  Decreased platelet count: 0.00  Increased blood bilirubin: 0.00  Elevated AST: 0.00 | Beta | Published literature | Direct medical costs: treatment, subsequent treatment, drug administration, management of AEs, follow-up, EOL care costs |

Abbreviations: AE, adverse event; ALT, alanine transaminase; AST, aspartate transaminase; BSC, best supportive care; DM, diabetes mellitus; EOL, end-of-life; HCC, hepatocellular carcinoma; NICE, National Institute for Health and Care Excellence; pCODR, pan-Canadian Oncology Drug Review; PD, progressive disease; PFS, progression-free survival; TA, technology appraisal; US, United States.

**References**

1. Cabibbo G, Celsa C, Enea M, Battaglia S, Rizzo GEM, Grimaudo S, et al. Optimizing sequential systemic therapies for advanced hepatocellular carcinoma: a decision analysis. Cancers. 2020;12(8):16.

2. Cabibbo G, Reig M, Celsa C, Torres F, Battaglia S, Enea M, et al. First-line immune checkpoint inhibitor-based sequential therapies for advanced hepatocellular carcinoma: rationale for future trials. Liver Cancer. 2022;11(1):75-84.

3. Cai H, Zhang L, Li N, Zheng B, Liu M. Lenvatinib versus sorafenib for unresectable hepatocellular carcinoma: a cost-effectiveness analysis. Journal of Comparative Effectiveness Research. 2020;9(8):553-62.

4. Camma C, Cabibbo G, Petta S, Enea M, Iavarone M, Grieco A, et al. Cost-effectiveness of sorafenib treatment in field practice for patients with hepatocellular carcinoma. Hepatology. 2013;57(3):1046-54.

5. Carr BI, Carroll S, Muszbek N, Gondek K. Economic evaluation of sorafenib in unresectable hepatocellular carcinoma. Journal of Gastroenterology and Hepatology (Australia). 2010;25(11):1739-46.

6. Chiang CL, Chan SK, Lee SF, Wong IOL, Choi HCW. Cost-effectiveness of pembrolizumab as a second-line therapy for hepatocellular carcinoma. JAMA Network Open. 2020;4(1):e2033761.

7. Chiang CL, Chan SK, Lee SF, Choi HC. First-line atezolizumab plus bevacizumab versus sorafenib in hepatocellular carcinoma: a cost-effectiveness analysis. Cancers (Basel). 2021;13(5).

8. Elsisi GH, Nada Y, Rashad N, Carapinha J. Cost-effectiveness of sorafenib versus best supportive care in advanced hepatocellular carcinoma in Egypt. Journal of Medical Economics. 2019;22(2):163-8.

9. Guan H, Wang C, Zhao Z, Han S. Cost-effectiveness of donafenib as first-line treatment of unresectable hepatocellular carcinoma in China. Advances in Therapy. 2022;39(7):3334-46.

10. Gupta N, Verma RK, Prinja S, Dhiman RK. Cost-effectiveness of sorafenib for treatment of advanced hepatocellular carcinoma in India. Journal of Clinical and Experimental Hepatology. 2019;9(4):468-75.

11. Ho JC, Hsieh ML, Chuang PH, Hsieh VC. Cost-effectiveness of sorafenib monotherapy and selected combination therapy with sorafenib in patients with advanced hepatocellular carcinoma. Value in Health Regional Issues. 2018;15:120-6.

12. Hou Y, Wu B. Atezolizumab plus bevacizumab versus sorafenib as first-line treatment for unresectable hepatocellular carcinoma: a cost-effectiveness analysis. Cancer Communications. 2020;40(12):743-5.

13. Ikeda S, Kudo M, Izumi N, Kobayashi M, Azuma M, Meier G, et al. Cost-effectiveness of lenvatinib in the treatment of patients with unresectable hepatocellular carcinomas in Japan: an analysis using data from Japanese patients in the REFLECT trial. Value in Health Regional Issues. 2021;24:82-9.

14. Kim JJ, McFarlane T, Tully S, Wong WWL. Lenvatinib versus sorafenib as first-line treatment of unresectable hepatocellular carcinoma: a cost-utility analysis. Oncologist. 2020;25(3):e512-e9.

15. Kobayashi M, Kudo M, Izumi N, Kaneko S, Azuma M, Copher R, et al. Cost-effectiveness analysis of lenvatinib treatment for patients with unresectable hepatocellular carcinoma (uHCC) compared with sorafenib in Japan. Journal of Gastroenterology. 2019;54(6):558-70.

16. Li M, Lin S, Wilson L, Huang P, Wang H, Lai S, et al. Cost-effectiveness analysis of hepatic arterial infusion of FOLFOX combined sorafenib for advanced hepatocellular carcinoma with portal vein invasion. Frontiers in Oncology. 2021;11.

17. Li L, Yang S, Chen Y, Tian L, He Y, Wu B, et al. Immune checkpoint inhibitors plus an anti-VEGF antibody as the first-line treatment for unresectable hepatocellular carcinoma: a network meta-analysis and cost-effectiveness analysis. Frontiers in Pharmacology. 2022;13:891008.

18. Li Y, Liang X, Li H, Chen X. Atezolizumab plus bevacizumab versus nivolumab as first-line treatment for advanced or unresectable hepatocellular carcinoma: a cost-effectiveness analysis. Cancer. 2022;128(22):3995-4003.

19. Liao W, Huang J, Hutton D, Zhu G, Wu Q, Wen F, et al. Cost-effectiveness analysis of cabozantinib as second-line therapy in advanced hepatocellular carcinoma. Liver International. 2019;39(12):2408-16.

20. Liu R, Qiu K, Jiang Y, Pang J. Economic evaluation of atezolizumab plus bevacizumab versus sorafenib in the first-line treatment of unresectable hepatocellular carcinoma. China Pharmacist. 2022;25(5):825-31.

21. Meng R, Zhou T, Shi F, Wang Z, Luo M, Ma A. Cost-utility analysis of pembrolizumab in the second-line treatment of advanced hepatocellular carcinoma based on two models. China Pharmacy. 2021;32(22):2761-6.

22. Meng R, Cao Y, Zhou T, Hu H, Qiu Y. The cost effectiveness of donafenib compared with sorafenib for the first-line treatment of unresectable or metastatic hepatocellular carcinoma in China. Frontiers in Public Health. 2022;10:794131.

23. Meng R, Zhang X, Zhou T, Luo M, Qiu Y. Cost-effectiveness analysis of donafenib versus lenvatinib for first-line treatment of unresectable or metastatic hepatocellular carcinoma. Expert Review of Pharmacoeconomics & Outcomes Research. 2022;22(7):1079-86.

24. Meyers BM, Vogel A, Marotta P, Kavan P, Kamboj L, Pan J, et al. The cost-effectiveness of lenvatinib in the treatment of advanced or unresectable hepatocellular carcinoma from a Canadian perspective. Canadian Journal of Gastroenterology and Hepatology. 2021;2021.

25. Muszbek N, Shah S, Carroll S, McDonald H, Dale P, Maroun J, et al. Economic evaluation of sorafenib in the treatment of hepatocellular carcinoma in Canada. Current Medical Research and Opinion. 2008;24(12):3559-69.

26. Parikh ND, Singal AG, Hutton DW. Cost effectiveness of regorafenib as second-line therapy for patients with advanced hepatocellular carcinoma. Cancer. 2017;123(19):3725-31.

27. Peng Y, Zeng X, Peng L, Liu Q, Yi L, Luo X, et al. Sintilimab plus bevacizumab biosimilar versus sorafenib as first-line treatment for unresectable hepatocellular carcinoma: a cost-effectiveness analysis. Frontiers in Pharmacology. 2022;13.

28. Qin S, Kruger E, Tan SC, Cheng S, Wang N, Liang J. Cost-effectiveness analysis of FOLFOX4 and sorafenib for the treatment of advanced hepatocellular carcinoma in China. Cost Effectiveness and Resource Allocation. 2018;16(1).

29. Saiyed M, Byrnes J, Srivastava T, Scuffham P, Downes M. Cost-effectiveness of lenvatinib compared with sorafenib for the first-line treatment of advanced hepatocellular carcinoma in Australia. Clinical Drug Investigation. 2020;40(12):1167-76.

30. Sangmala P, Lamlertthon W, Siri P, Jaroenpatarapesaj S. Economic evaluation of sorafenib treatment of patients with advanced hepatocellular carcinoma at Chulabhorn Hospital. Journal of the Medical Association of Thailand. 2018;101(6):S171-S83.

31. Sherrow C, Attwood K, Zhou K, Mukherjee S, Iyer R, Fountzilas C. Sequencing systemic therapy pathways for advanced hepatocellular carcinoma: a cost effectiveness analysis. Liver Cancer. 2020;9(5):549-62.

32. Shi F, Meng R, Wang Z, Rui M, Shang Y, Ma A. Cost-effectiveness analysis of applying camrelizumab as second-line therapy for the treatment of advanced hepatocellular carcinoma. Chinese Health Economics. 2021;40(2):62-5.

33. Shlomai A, Leshno M, Goldstein DA. Regorafenib treatment for patients with hepatocellular carcinoma who progressed on sorafenib-A cost-effectiveness analysis. PLoS One. 2018;13(11).

34. Shlomai A, Leshno M, Goldstein DA. Cabozantinib for patients with advanced hepatocellular carcinoma: a cost-effectiveness analysis. Therapeutic Advances in Gastroenterology. 2019;12:1756284819878304.

35. Sieg M, Hartmann M, Settmacher U, Arefian H. Comparative cost-effectiveness of cabozantinib as second-line therapy for patients with advanced hepatocellular carcinoma in Germany and the United States. BMC Gastroenterology. 2020;20(1).

36. Soto-Perez-de-Celis E, Aguiar PN, Cordon ML, Chavarri-Guerra Y, Lopes GD. Cost-effectiveness of cabozantinib in the second-line treatment of advanced hepatocellular carcinoma. Journal of the National Comprehensive Cancer Network: JNCCN. 2019;17(6):669-75.

37. Su D, Wu B, Shi LZ. Cost-effectiveness of atezolizumab plus bevacizumab vs sorafenib as first-line treatment of unresectable hepatocellular carcinoma. JAMA Network Open. 2021;4(2):11.

38. Wen F, Zheng H, Zhang P, Liao W, Zhou K, Li Q. Atezolizumab and bevacizumab combination compared with sorafenib as the first-line systemic treatment for patients with unresectable hepatocellular carcinoma: a cost-effectiveness analysis in China and the United states. Liver International. 2021;41(5):1097-104.

39. Zhang P, Yang Y, Wen F, He X, Tang R, Du Z, et al. Cost-effectiveness of sorafenib as a first-line treatment for advanced hepatocellular carcinoma. European Journal of Gastroenterology and Hepatology. 2015;27(7):853-9.

40. Zhang P, Wen F, Li Q. FOLFOX4 or sorafenib as the first-line treatments for advanced hepatocellular carcinoma: a cost-effectiveness analysis. Digestive and Liver Disease. 2016;48(12):1492-7.

41. Zhang X, Wang J, Shi J, Jia X, Dang S, Wang W. Cost-effectiveness of atezolizumab plus bevacizumab vs sorafenib for patients with unresectable or metastatic hepatocellular carcinoma. JAMA Network Open. 2021;4(4):e214846.

42. Zhao M, Pan X, Yin Y, Hu H, Wei J, Bai Z, et al. Cost-effectiveness analysis of five systemic treatments for unresectable hepatocellular carcinoma in China: an economic evaluation based on network meta-analysis. Frontiers in Public Health. 2022;10:869960.

43. Zheng H, Qin Z, Qiu X, Zhan M, Wen F, Xu T. Cost-effectiveness analysis of ramucirumab treatment for patients with hepatocellular carcinoma who progressed on sorafenib with α-fetoprotein concentrations of at least 400 ng/ml. Journal of Medical Economics. 2020;23(4):347-52.

44. Zhou T, Cao Y, Wang X, Yang L, Wang Z, Ma A, et al. Economic evaluation of sintilimab plus bevacizumab versus sorafenib as a first-line treatment for unresectable hepatocellular carcinoma. Advances in Therapy. 2022;39(5):2165-77.

45. Zhou T, Wang X, Cao Y, Yang L, Wang Z, Ma A, et al. Cost-effectiveness analysis of sintilimab plus bevacizumab biosimilar compared with lenvatinib as the first-line treatment of unresectable or metastatic hepatocellular carcinoma. BMC Health Services Research. 2022;22(1):1367.

46. National Institute for Health and Care Excellence (NICE). NICE TA474. Sorafenib for treating advanced hepatocellular carcinoma. 2017.

47. National Institute for Health and Care Excellence (NICE). NICE TA551. Lenvatinib for untreated advanced hepatocellular carcinoma. 2018.

48. National Institute for Health and Care Excellence (NICE). NICE TA555. Regorafenib for previously treated advanced hepatocellular carcinoma. 2019.

49. National Institute for Health and Care Excellence (NICE). NICE TA666. Atezolizumab with bevacizumab for treating advanced or unresectable hepatocellular carcinoma. 2020.

50. National Institute for Health and Care Excellence (NICE). NICE TA849. Cabozantinib for previously treated advanced hepatocellular carcinoma. 2022.

51. pan-Canadian Oncology Drug Review (pCODR). pCODR 10119. Regorafenib for treatment of patients with unresectable hepatocellular carcinoma (HCC) who have been previously treated with sorafenib. 2018.

52. pan-Canadian Oncology Drug Review (pCODR). pCODR 10134. Nivolumab for the treatment of adult patients with advanced (not amenable to curative therapy or local therapeutic measures) or metastatic hepatocellular carcinoma who are intolerant to or have progressed on sorafenib therapy. 2018.

53. pan-Canadian Oncology Drug Review (pCODR). pCODR 10175. Lenvatinib for the first-line treatment of adult patients with unresectable hepatocellular carcinoma (HCC). 2019.

54. pan-Canadian Oncology Drug Review (pCODR). pCODR 10186. Cabozantinib for the treatment of hepatocellular carcinoma (HCC) in adults after prior therapy. 2020.

55. pan-Canadian Oncology Drug Review (pCODR). pCODR 10217. Atezolizumab in combination with bevacizumab for the first-line treatment of adult patients with unresectable or metastatic hepatocellular carcinoma (HCC) who require systemic therapy. 2020.
